# Structures of nucleotide-bound human telomerase at several steps of its telomeric DNA repeat addition cycle

**DOI:** 10.1038/s41467-026-68560-8

**Published:** 2026-01-21

**Authors:** Sebastian Balch, Elsa Franco-Echevarría, George E. Ghanim, Rachael C. Kretsch, Rhiju Das, Thi Hoang Duong Nguyen

**Affiliations:** 1 Medical Research Council Laboratory of Molecular Biology, Francis Crick Avenue, Cambridge, UK; 2 Biophysics Program, Stanford University, Stanford, CA, USA; 3 Department of Biochemistry, Stanford University, Stanford, CA, USA; 4 Department of Physics, Stanford University, Stanford, CA, USA; 5 Howard Hughes Medical Institute, Stanford, CA, USA; 6 Present Address: Department of Molecular Biology, Princeton University, Princeton, NJ USA

**Keywords:** Cryoelectron microscopy, RNA, DNA

## Abstract

In most eukaryotes, the reverse transcriptase telomerase counteracts telomere shortening by processively adding telomeric DNA repeat sequences to chromosome ends. Telomerase activity depends on the telomerase reverse transcriptase (TERT) and the telomerase RNA (hTR in humans). Processive telomere elongation is critical for genome stability, and defects in this mechanism are linked to cellular dysfunction and human disease. However, the structural basis for telomerase repeat addition processivity in humans has remained elusive. Here, we present cryo-electron microscopy structures of human telomerase bound to telomeric DNA and an incoming nucleotide, captured at three distinct stages of its repeat addition cycle: initiation, elongation, and pre-termination. Across these states, the TERT active site maintains a conserved architecture that stabilises a short DNA–RNA duplex of constant length of four base-pairs. Beyond the active site, we identify dynamic structural features in both TERT and hTR that facilitate substrate engagement and RNA template repositioning, thereby supporting the synthesis of successive telomeric repeats. Together, these structures provide key insights into how human telomerase achieves its unique processivity to maintain telomere length and genome integrity.

## Abstract (web summary)

To maintain genome stability, the specialised reverse transcriptase telomerase processively adds telomeric DNA repeats to chromosome ends. Here the authors reported structures of human telomerase at three stages of telomeric repeat synthesis, providing key insights into its mechanism.

## Introduction

Telomerase is a specialised reverse transcriptase (RTs) that synthesises the G-rich telomeric repeats (GGTTAG_n_ in humans) at the end of eukaryotic chromosomes^1^. In humans, the resulting telomeric repeats are bound by shelterin, a protein complex that protects chromosome ends from unwanted DNA damage responses^2^. High-fidelity synthesis of telomeric repeats by telomerase is critical for proper shelterin function and, consequently, genome stability. Dysregulation of telomerase is implicated in human disease: its aberrant activation is observed in ~90% of cancers, whereas loss-of-function mutations are linked to several premature aging syndromes^3,4^.

**Fig. 1:**
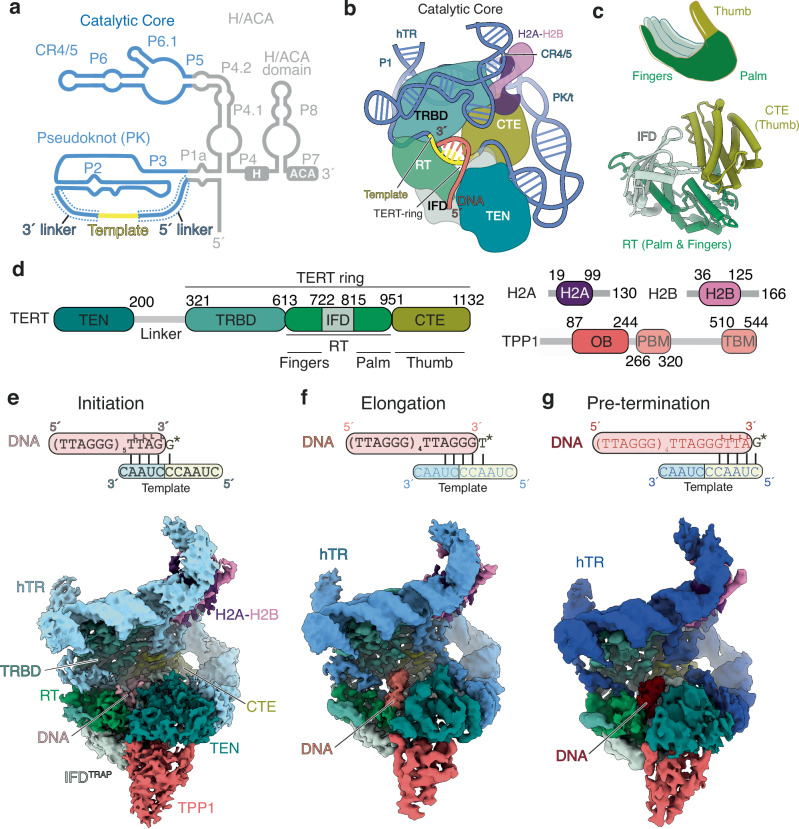
Cryo-EM structures of the human telomerase catalytic core at three different RAP states. **a** Secondary structure schematic of hTR. Regions in the catalytic core and H/ACA RNP are colored blue and grey, respectively. The template region is highlighted in yellow. CR4/5 conserved regions 4 and 5; PK pseudoknot. **b** Schematic of the catalytic core of human telomerase. TERT telomerase reverse transcriptase; TEN telomerase essential N-terminal domain; TRBD telomerase RNA binding domain; RT reverse transcriptase; IFD (also IFD^TRAP^) insertion in the fingers subdomain; CTE C-terminal extension. **c** Schematic (top) and structure (bottom) of the conserved DNA polymerase right-hand architecture of TERT. **d** Domain schematics of proteins subunits in the human telomerase catalytic core. OB oligosaccharide/oligonucleotide binding domain; PBM POT1 binding motif; TBM TIN2 binding motif. The colour scheme of proteins and domains shown is used in the remaining panels unless otherwise stated. **e** 3.5-Å cryo-EM map of the telomerase catalytic core with the initiation DNA substrate and the non-hydrolysable dGTP analog dGpCpp (denoted as G* in the top schematic). **f** 3.2-Å cryo-EM map of the telomerase catalytic core with the elongation DNA substrate and the non-hydrolysable dTTP analog dTpCpp (denoted as T* in the top schematic). **g** 3.8-Å cryo-EM map of the telomerase catalytic core with the pre-termination DNA and the non-hydrolysable dGTP analog dGpCpp (denoted as G* in the top schematic).

Telomeric DNA synthesis requires two core components: the telomerase reverse transcriptase (TERT) and an RNA template within telomerase RNA (hTR in humans)^5^. Human telomerase is organised into two functionally distinct modules connected by hTR: a catalytic core and an H and ACA box (H/ACA) ribonucleoprotein (RNP)^6^ (Fig. 1a). The catalytic core includes TERT, two domains of hTR, the pseudoknot/template (PK/t) and conserved regions 4 and 5 (CR4/5), and a histone H2A-H2B dimer^7^ (Fig. 1b). The H/ACA RNP consists of two H/ACA heterotetramers and the telomerase Cajal body localisation factor TCAB1, all of which bind to the H/ACA domain of hTR. While the catalytic core carries out telomeric DNA synthesis, the H/ACA RNP is required for telomerase biogenesis and localisation^8–10^.

TERT comprises four domains: the telomerase essential N-terminal (TEN) domain, the telomerase RNA binding domain (TRBD), the reverse transcriptase (RT) domain and the C-terminal extension (CTE) (Fig. 1b–d). The RT domain and CTE adopt a canonical DNA polymerase right-hand architecture with the palm and fingers (RT) and the thumb (CTE) (Fig. 1c). The TRBD binds hTR with high-affinity and, together with the RT domain and CTE, form the “TERT-ring” that encloses the DNA-RNA duplex^11,12^ (Fig. 1b, d). The RT domain contains a telomerase-specific insertion in the fingers subdomain (IFD), also known as the TRAP motif^13,14^. Hereafter, we refer to this insertion as IFD^TRAP^ (Fig. 1d). The TEN domain and the IFD^TRAP^ interact with each other to stabilise the 5′ end of the DNA substrate positioned outside the TERT-ring^15^ (Fig. 1b).

Unlike conventional retroviral RTs, telomerase exhibits a unique property known as repeat addition processivity (RAP), enabling it to add multiple telomeric repeats to a DNA substrate during a single binding event^5,16^. It is thought that RAP is achieved through a dynamic cycle that allows iterative reuse of the RNA template within hTR^5^. This RAP cycle can be described in four stages: initiation, elongation, termination and translocation (Supplementary Fig. 1a). The RNA template is organised into two functional segments: an alignment region and a templated region. Repeat synthesis initiates when the 3′ GGTTAG telomeric repeat base-pairs with the alignment region of the template. Following six consecutive nucleotide addition using the templated region, synthesis terminates. The DNA–RNA duplex dissociates, and the telomeric DNA realigns back to the alignment region for the next round of repeat synthesis (Supplementary Fig. 1a). RAP is crucial for telomere maintenance in vivo, and its disruption either by chemical inhibition or disease-linked mutations leads to telomere shortening and cellular dysfunction^17–21^. Therefore, elucidating the molecular basis of RAP is of significant biological and clinical importance.

Prior biochemical and genetic studies of human telomerase RAP have often relied on structural insights from the flour beetle *Tribolium castaneum* TERT^5,12,22^. However, *Tribolium* telomerase RNA has not been identified, and its TERT lacks the TEN domain and the IFD^TRAP^ that have been shown to be essential for RAP in the human enzyme^13,23^. Available human telomerase structures have captured the enzyme in an elongation state, using a DNA substrate ending in a TTAGGG permutation (Supplementary Fig. 1a)^7,15,24,25^. On the other hand, cryo-electron microscopy (cryo-EM) studies of *Tetrahymena thermophila* telomerase have visualised multiple steps of the RAP cycle, revealing key features such as the 5′ and 3′ boundaries of the RNA template and mechanisms for coordinating the DNA–RNA duplex during repeat synthesis^26^. Yet, due to the significant evolutionary divergence of TERT, TER, and telomeric repeat sequences among species^27^, these findings cannot be fully extrapolated to the human system. As a result, the detailed mechanism of the human telomerase RAP cycle has remained elusive.

To address this, we present three cryo-EM structures of human telomerase bound to an incoming nucleotide and telomeric DNA substrates, capturing the enzyme in the initiation, elongation and pre-termination states of the RAP cycle. These structures uncover conserved as well as human-specific features in TERT and hTR that are critical for RAP. Together, they provide a mechanistic framework for how human telomerase achieves repeat addition processivity.

## Results

### Structure determination of incoming nucleotide-bound human telomerase at three distinct states of the RAP cycle

We reconstituted human telomerase by overexpression of TERT and hTR in human cells, followed by a two-step purification^6,28^ (Supplementary Fig. 1b). In the second purification step, Strep-tagged telomerase was immobilised on Strep resin for assembly with telomeric DNA substrates mimicking the initiation, elongation, and pre-termination stages of the catalytic cycle (Supplementary Fig. 1b–e, 2a–c). Upon elution from Strep resin, the presence of the bound DNA was verified by activity assays, in which the corresponding DNA substrate was omitted (Supplementary Fig. 2a–c). DNA substrates corresponding to the initiation and pre-termination states have significantly higher dissociation rates compared to the elongation substrate ending in GGG^29^. Therefore, to stabilise these weaker complexes, we employed three strategies.

First, the TPP1–POT1–TIN2 (TPT) shelterin subcomplex has been shown to stabilise TERT–DNA interactions^15,30^. We included TPT during reconstitution of all complexes (Supplementary Fig. 1b–e, 2a–c). Second, we incorporated four locked nucleic acid (LDNA) analogues at the 3′ end of the initiation and pre-termination substrates (Supplementary Fig. 2a, c). The 2′-oxygen and the 4′-carbon of the ribose moiety in locked nucleotides are bridged, thereby reducing its conformation flexibility and enhancing duplex stability^31,32^ (Supplementary Fig. 2d). Third, we added a non-hydrolysable dNTP analogue of the incoming nucleotide (dGpCpp for initiation and pre-termination; and dTpCpp for elongation) to further stabilise DNA binding. Consistent with this, the addition of the incoming nucleotide enhanced substrate retention in complexes assembled with unmodified DNA (Supplementary Fig. 1c–e, lanes 2 and 3). Furthermore, available structures of human telomerase lack an incoming nucleotide^7,15,24,25^. The inclusion of the incoming nucleotide in the reconstitution would allow us to capture human telomerase in a nucleotide-bound state.

The composition of the telomerase–DNA–TPT complex was verified by SDS-PAGE and negative stain electron microscopy (EM) (Supplementary Fig. 2a–c, e–g). DNA extension products were observed in telomerase activity assays, which lack DNA or LDNA substrates (Supplementary Fig. 2a, lane 3; b, lane 2; c, lane 3), confirming the retention of the DNA substrates. DNA substrates containing LDNAs supported minimal RAP activity, likely due to reduced product release resulting from increased duplex stability (Supplementary Fig. 2a, c).

We then used cryo-EM to determine structures of telomerase–TPT complexes assembled with each telomeric substrate (Supplementary Fig. 3a–f). Previous work has shown that the catalytic core, but not the H/ACA RNP, is involved in DNA binding (Fig. 1b)^7,15,33^. Thus, to address the conformational flexibility of these two lobes, we collected large datasets and applied signal subtraction to isolate only the catalytic core, followed by focused classification of the active site (Supplementary Fig. 3g–i, 4). This yielded reconstructions of the telomerase catalytic cores bound to DNA and an incoming dNTP at 3.5 Å (initiation), 3.2 Å (elongation), and 3.8 Å (pre-termination) resolution (Fig. 1e–g, Supplementary Fig. 4, 5, 6, Supplementary Table 1). While TPP1 was visible in all three maps (Fig. 1e–g), POT1 and TIN2 were unresolved due to their flexibility, as previously reported^15^. POT1 has been shown to bind to the more distal 5′ region of the DNA and did not influence the positioning of the 3′ end in the active site of telomerase^15^. Each reconstruction revealed clear density for both the telomeric DNA and the incoming nucleotide (Supplementary Fig. 6a–c), enabling us to model human telomerase catalytic cores at three distinct states of the RAP cycle.

### Structures of telomerase in three distinct states of its RAP cycle reveal a constant duplex length of 4 or 5 base-pairs

Across the three states of the telomerase RAP cycle, the catalytic core of telomerase maintains a similar global architecture (Fig. 1b, e–g, Supplementary Fig. 5e, j, o). The PK/t and CR4/5 domain of hTR encircle TERT, while the TERT-ring accommodates the DNA–RNA duplex formed between the hTR template and the telomeric DNA (Fig. 1b, Supplementary Fig. 5e, j, o). The TEN domain and IFD^TRAP^, located outside of the TERT-ring, bind the DNA and RNA template regions distal from the duplex-binding site (Fig. 1b, e–g, Supplementary Fig. 5e, j, o). They exhibit increased flexibility compared to the TERT-ring, reflected by their lower local resolution in cryo-EM maps (Supplementary Fig. 5d, i, n). As observed previously, the CR4/5 also binds a histone H2A–H2B dimer^7^, and TPP1 associates with TERT^15,25^ (Fig. 1e–g, Supplementary Fig. 5e, j, o).

**Fig. 2:**
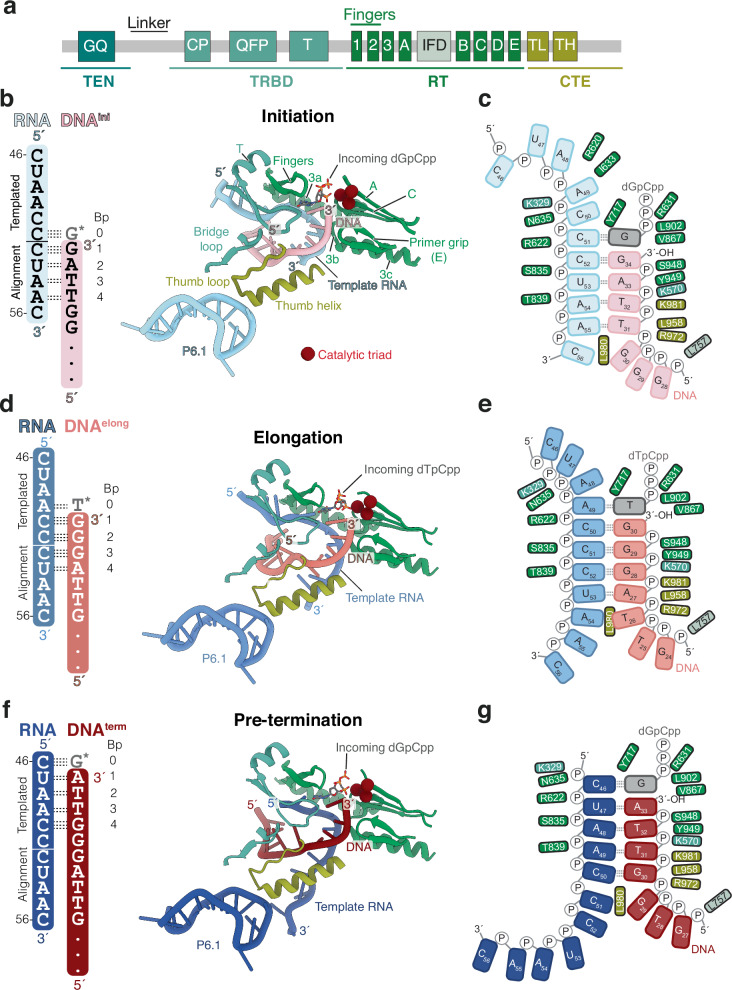
A four base-pair DNA–RNA duplex is maintained throughout the catalytic cycle with minimal changes in conserved TERT motifs. **a** Domain schematic of TERT with conserved motifs important for telomerase activity shown. CP conserved protein; IFD (also IFD^TRAP^) insertion in the fingers subdomain; TL thumb loop; TH thumb helix; TEN telomerase essential N-terminal domain; TRBD telomerase RNA binding domain; RT reverse transcriptase; CTE C-terminal extension. **b**, **d**, **f** Schematic of DNA–RNA duplex (left panel) and active sites showing the hTR template region, DNA substrate, incoming dNTP, and conserved motifs within the TERT-ring (right panel) for the initiation complex (**b**), elongation complex (**d**), and pre-termination complex (**f**). Motifs are coloured according to the TERT domain they belong to as shown in (**a**). **c**, **e**, **g** Schematics of the DNA–RNA duplex observed in the initiation complex (**c**), elongation complex (**e**), and pre-termination complex (**g**). Incoming dNTP is represented in grey and the interactions with TERT residues are shown. The domains of TERT are coloured as shown in (**a**).

Prior human telomerase structures in the elongation state showed a 4 base-pair duplex in the absence of an incoming nucleotide^7,15,24,25^. How the length of the DNA–RNA duplex changes during RAP in human telomerase remains unclear. Based on base complementarity, the duplex can theoretically extend to 10 or 11 base pairs as synthesis progresses toward the termination stage^34–36^ (Supplementary Fig. 1a). Strikingly, our structures reveal a consistent 4 base-pair DNA–RNA duplex in all three RAP states (Fig. 2a–g). In each case, the incoming dNTP is resolved in the active site of telomerase and base-paired with the template, extending the duplex to 5 base-pairs (Fig. 2a–g). The observed duplex length would explain why telomerase extends DNA substrate in a 5 base-pair DNA–RNA hybrid in *trans* more efficiently than longer duplexes^36^. Our data suggest that during repeat synthesis, human telomerase maintains a constant duplex of 4 or 5 base-pairs, depending on whether the incoming nucleotide is present. Each nucleotide addition is thus coupled with the melting of a base pair at the distal end of the duplex, preserving its overall length.

Notably, residues L980 in the CTE domain of TERT consistently marks the duplex terminus in all structures, supporting its proposed role as a “zipper head” that limits duplex length^24^ (Fig. 2c, e, g). Maintaining a short 4 base-pair duplex likely facilitates efficient strand melting during the translocation step of the RAP cycle. A similar duplex length has been reported in multiple structures of *Tetrahymena* telomerase bound to various DNA substrates^26^. Chemical probing of the RNA template in yeast telomerase also supported an invariant duplex length during repeat synthesis^37^. Together, these findings suggest that maintaining a short, constant duplex may be a conserved feature of telomerases across eukaryotes.

### Conserved active site motifs in TERT stabilise the DNA–RNA duplex and the incoming nucleotide

In the cryo-EM structures of all three catalytic states, we observed highly conserved interactions between the DNA–RNA duplex region and specific structural motifs within the TERT-ring (Fig. 2a, b, d, f). For simplicity, we define the base pair formed by the incoming dNTP and the RNA template as position 0, followed by base-pairs 1 through 4 in the 3′ to 5′ direction along the DNA strand (Fig. 2b, d, f, Supplementary Fig. 6a-c). The incoming dNTP and the two 3′-terminal nucleotides of the DNA (positions 0 to 2) are coordinated by motifs A and C, which contain the three essential catalytic aspartates, and motif E, known as the primer grip (Fig. 2b, d, f). The 5′-terminal two base-pairs of the duplex (positions 3 and 4) are further stabilised by the thumb helix, thumb loop, and motif T. On the RNA side, the duplexed region of the hTR template is clamped by motif 3 (residues 654–699), which is specific to telomerase and has been shown to be important for RAP^38^.

These structural observations align with previous mutational studies. Substitution of TERT residues involved in duplex binding impairs nucleotide addition and repeat addition processivity^19,20,25,34,36,38–45^ (see Supplementary Data 1 for a list of mutations). Interactions between TERT and the DNA–RNA duplex are conserved across the initiation, elongation, and pre-termination states, underscoring their critical role in duplex stabilisation and processive telomeric repeat synthesis.

Our structures also provide molecular insights into how telomerase coordinates the incoming nucleotide within its active site (Supplementary Fig. 6a–c). Compared to prior structures of telomerase in the elongation state without a bound nucleotide^7,15^, we observe that the fingers domain, motifs A and C, and the loop preceding the steric gate residue Y717 close around the incoming nucleotide (Supplementary Fig. 7a–c). In both the initiation and pre-termination states, the incoming dGpCpp nucleotide engages in strong stacking interactions with the 3′-terminal base of the DNA strand (Supplementary Fig. 6a, c). This stacking interaction of the incoming dGpCpp, together with base-pairing to the RNA template, likely accounts for the enhanced retention of the initiation and pre-termination DNA substrates upon addition of dGpCpp during reconstitution (Supplementary Fig. 1c, e).

Notably, increased concentrations of dGTP have been shown to enhance telomerase processivity and compensate for RAP defects caused by mutations in either the template alignment region or TERT residues involved in DNA–RNA duplex interactions^34,46,47^. dGTP is the first nucleotide incorporated into telomeric DNA following template realignment (Supplementary Fig. 1a). A prior single-molecule study has shown that this first dGTP incorporation is rate-limiting in repeat synthesis^48^. Therefore, the observed stabilisation by incoming dGTP likely promotes efficient realignment, contributing directly to RAP.

### Sequence identity of the DNA–RNA duplex influences its stability

Despite a constant duplex length and conserved protein-duplex interactions, telomerase displays differential affinities for the six possible telomeric permutations^29^. Substrates ending with TTAGGG (elongation state) dissociate more slowly, whereas those ending with GGGTTA (pre-termination state) dissociate more rapidly^29^. The underlying molecular basis for these differences remains poorly understood. To address this, we examined the conformations of the DNA–RNA duplex in our three structures.

In the initiation and pre-termination states, three of the four base-pairs in the duplex are weaker rA–dT pairs, whereas in the elongation state, the duplex contains three stronger rC–dG base-pairs (Fig. 2b, d, f), consistent with its greater duplex stability. Beyond base-pair composition, we also observed state-dependent differences in base-pairing geometry, particularly at positions distal to the active site.

In the initiation state, base-pairs at positions 1 and 2 adopt stable Watson–Crick–Franklin geometry, whereas base-pairs 3 and 4 are visibly distorted (Fig. 2c, and Supplementary Fig. 6a). In the elongation state, base-pairs 1 to 3 form stable rC–dG pairs, but the terminal rA–dT base pair at position 4 is distorted, as also reported previously^24^ (Fig. 2e, and Supplementary Fig. 6b). In the pre-termination state, distortions emerge as early as position 2 and are present in all subsequent positions (Fig. 2g, Supplementary Fig. 6c). These structural differences offer a mechanistic explanation for the high affinity of the 5′-TTAGGG-3′ permutation and the low affinity of the 5′-GGGTTA-3′ permutation for telomerase. Together, our findings indicate that DNA–RNA duplex stability changes during the repeat synthesis cycle and is influenced by the sequence identity of the DNA substrate.

### The 5′ and 3′ regions flanking the RNA template contribute differently to telomerase activity

Although the DNA–RNA duplex length within the active site remains constant, the RNA template in hTR must translocate through the reverse transcriptase active site of telomerase to support repeat synthesis. In *Tetrahymena* telomerase, the single-stranded RNA (ssRNA) regions flanking the template, termed the template boundary element (TBE) and template recognition element (TRE), are crucial for setting the 5′ and 3′ template boundaries, respectively^49–51^ (Supplementary Fig. 7d, right panel). The equivalent regions in hTR have diverged substantially from those in *Tetrahymena*^5,52^ (Supplementary Fig. 7d, left panel). Although these regions have been suggested to contribute to human telomerase activity by previous biochemical work^53^, the molecular mechanism of their function in repeat synthesis was unclear.

**Fig. 3:**
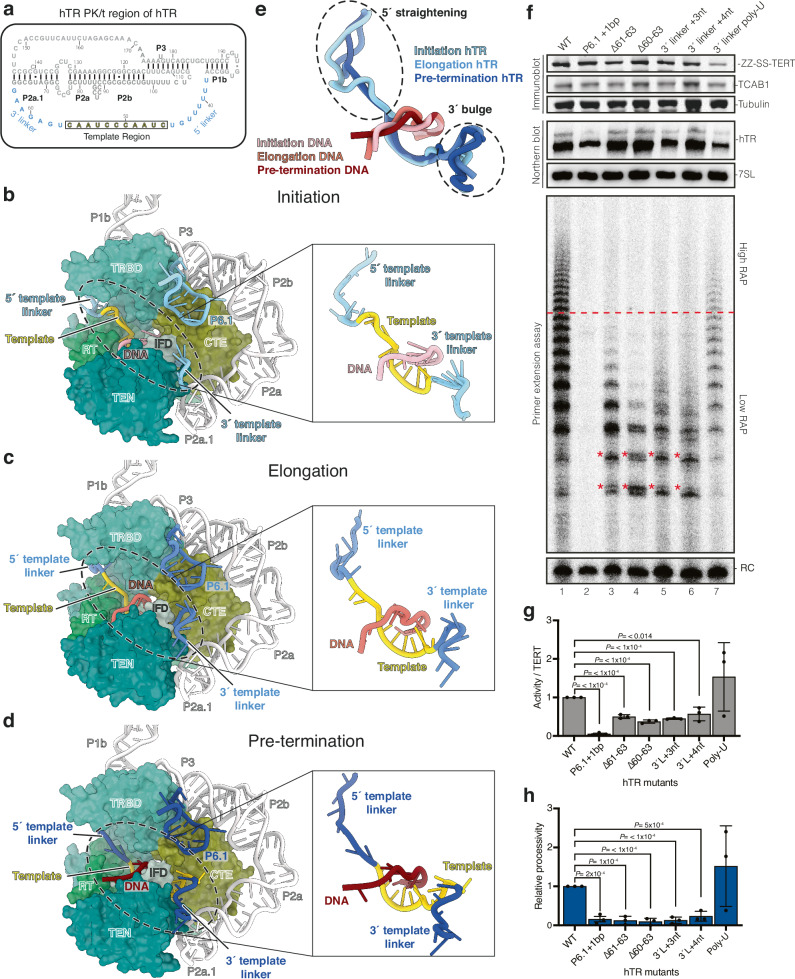
Dynamic transition of the RNA template and flanking regions through the telomerase active site during repeat synthesis. **a** Sequence and secondary structure of the pseudoknot/template (PK/t) domain of hTR. The RNA template and the 5′ template linker (upstream of the template) and 3′ template linker (downstream of the template) (nucleotides 38–63) are coloured as indicated, whereas the rest of the domain is coloured in grey. **b**–**d** Telomerase catalytic cores at the initiation (**b**), elongation (**c**), and pre-termination (**d**) states. TERT is shown in surface representation using the same colour scheme as in Fig. 1d. hTR and DNA substrates are shown in cartoon representation with insets highlighting the differences in the conformations of the 5′ and 3′ template linkers across the three reported states. TEN telomerase essential N-terminal domain; *TRBD* telomerase RNA binding domain; *RT* reverse transcriptase; IFD (also IFD^TRAP^) insertion in the fingers subdomain; CTE C-terminal extension. **e** Superposition of the RNA template and the 5′ and 3′ template linkers of hTR and the DNA substrates to show the difference in hTR linker conformations. **f** The effects of the 3′ template linker hTR mutations on telomerase activity and processivity. See Supplementary Fig. 8 h for schematics of the mutants. Wild-type (WT) and mutant telomerase were characterised using immunoblotting, Northern blotting, and telomerase activity assay (see Source Data for uncropped blots and gels with size markers). The red line across the activity assay denotes the boundary of low repeat addition processivity (RAP) (below) and high RAP (above) extension products. Differences in band pattern of mutants are highlighted with a red star. RC recovery control; bp basepair; nt nucleotide. **g** Quantification of telomerase activity relative to the TERT expression levels of WT and hTR mutant telomerase in f. TERT levels were normalised to tubulin levels, and activity was normalised to recovery control signal. Activity/TERT levels were calculated using these normalised values; and mutant activity/TERT levels were normalised to the WT level. **h** Quantification of relative processivity levels of the WT and hTR mutant telomerase shown in f. Relative processivity was calculated by dividing activity at high RAP (above red line) by low RAP (below red line). Values were normalised to WT. Experiments shown in f and subsequently quantified in g and h were performed in biological triplicate (see Source Data for replicate data). Data shown in g and h were quantified using a two-tailed *t* test. Data are presented as the mean values and error bars represent the standard deviation. Significant *P* values are reported. Source data are provided as a Source Data file.

We observed substantial structural rearrangements in the template 5′ and 3′ flanking ssRNA regions across the captured catalytic states (Fig. 3a–e). Henceforth, we refer to these regions as the 5′ and 3′ template linkers (nucleotides 38–45 and 57–63, respectively; Fig. 3a). These linkers exhibit high flexibility in all three maps, as indicated by local resolution analysis (Supplementary Figs. 5d, i, n, 8a–c). To sample their conformational heterogeneity, we applied DRRAFTER modeling^54^ to generate ensembles for these linkers alongside the consensus structure (Supplementary Fig. 8d–f and Supplementary Data 2–4).

As telomerase progresses from the initiation to the pre-termination state, the 5′ template linker, analogous to the *Tetrahymena* TBE, shortens as the RNA template is threaded through the active site (Fig. 3b–e). The 5′ template linker region is anchored by the adjacent G–C-rich P1 stem, which defines the 5′ template boundary in humans^27,55^ (Fig. 3a). Across all three structures, the P1 stem remains stable, while the 5′ template linker becomes fully stretched in the pre-termination state (Fig. 3b–e). Previous biochemical studies have shown that deletion or extension of the 5′ linker impairs activity and leads to boundary bypass, although RAP remains unaffected^53^. Together, these observations suggest that the 5′ linker acts as a flexible tether, akin to a bungee cord, allowing the template to feed into the active site while maintaining connection to the fixed P1 stem. Upon reaching the 5′ end of the template, this tether becomes maximally extended, enforcing termination of repeat synthesis (Fig. 3d, e).

In contrast, the 3′ template linker, analogous to the *Tetrahymena* TRE, lengthens during repeat synthesis. In our structures, the extended 3′ template linker in the pre-termination state forms a bulge and projects toward the P6.1 stem-loop of the CR4/5 domain (Fig. 3d, e). In *Tetrahymena* telomerase, the 3′ template linker traverses a narrow, positively charged tunnel formed by the thumb helix and IFD^TRAP^, which prevents the template RNA from moving backwards^26^ (Supplementary Fig. 7f). The human enzyme lacks such a defined tunnel, and the 3′ template linker in human telomerase takes a different path from that in *Tetrahymena* (Supplementary Fig. 7e, f, middle panels). Instead, steric constraints arise from the proximity of the thumb helix, IFD^TRAP^, and the P6.1 stem-loop (Fig. 3d, and Supplementary Fig. 7e, middle panel), implying distinct modes of 3′ template linker regulation between species.

To assess the importance of the 3′ template linker for RAP, we reconstituted human telomerase variants with deletions or insertions in this region (Fig. 3f, and Supplementary Fig. 8h). Deletion of two or three nucleotides (nucleotides 60–63) reduced RAP and altered the pattern of extension products, likely reflecting impaired template positioning or premature release of incomplete repeats (Fig. 3f, lanes 3 and 4; 3 g, h). Conversely, insertion of three or four nucleotides also markedly decreased RAP, though changes in product patterns were less pronounced (Fig. 3f, lanes 4 and 6; 3 g, h). These results indicate that human telomerase cannot accommodate significant alterations in 3′ template linker length without compromising processivity.

To evaluate the role of sequence specificity in the 3′ template linker, we reconstituted telomerase with all nucleotides in the 3′ template linker mutated to uracil. This mutant maintained comparable telomerase activity and RAP per TERT molecule (Fig. 3f, lane 7; 3 g, h), suggesting that the linker length, rather than sequence, is critical for function. Consistent with this, prior biochemical work with telomerase reconstituted in rabbit reticulocyte lysate also demonstrated that changes in linker length, but not sequence, affect activity and RAP^35,53^. Our structural data support the interpretation that no base-specific contacts are observed between the 3′ linker and TERT in all our structures. Nonetheless, reduced levels of TERT and hTR in the poly-U mutant imply possible defects in RNP assembly or RNA stability (Fig. 3f, lane 7; 3 g, h).

Upon reaching the pre-termination state, the bulged 3′ template linker moves towards the P6.1 stem-loop of hTR CR4/5 domain (Fig. 3d). Further extension of the linker would create a steric clash with this structural element. A prior study showed that extending the P6.1 stem-loop by four base pairs abolished telomerase activity^56^. Consistently, our P6.1 stem-loop mutant containing an additional C–G base-pair also eliminated activity (Fig. 3f, lane 2; 3 g, h; Supplementary Fig. 8h). Structural analysis reveals that both the stem and terminal loop of P6.1 form extensive contacts with the thumb loop and thumb helix, which in turn stabilise the DNA–RNA duplex (Fig. 2b, d, f, and Supplementary Fig. 7e, middle panel). These interactions are preserved throughout all three reported states (Fig. 2b, d, f), highlighting their importance for processivity. Disruption of either the stem or loop region of P6.1 would destabilise the duplex by weakening its interactions with the thumb helix and thumb loop. Our findings thus provide a structural explanation for why the CR4/5 domain, particularly the P6.1 stem-loop, is essential for telomerase function^18,56–59^.

### The IFD^TRAP^ and TEN domain of TERT affect telomerase activity and processivity through interactions with both DNA and RNA

**Fig. 4:**
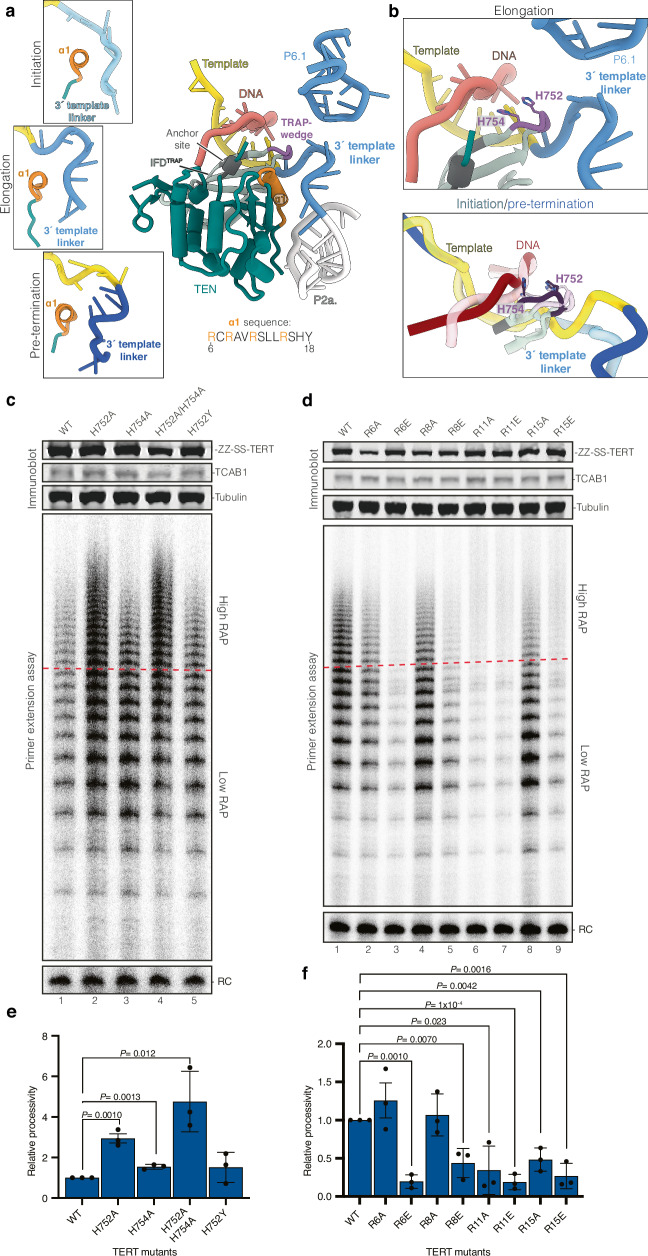
Helix α1 of TERT and IFD TRAP-wedge influence RAP through interaction with the 3′ template linker. **a** Cartoon representation of the TEN domain and IFD^TRAP^ of TERT with the DNA substrate, 3′ template linker, the P2a.1 stem, and P6.1 stem of the CR4/5 domain of hTR. Cartoon shows only the elongation state for simplicity. The three insets on the left show the interaction between helix α1 of the TEN domain and the 3′ template linker in the initiation, elongation, and pre-termination states. TEN telomerase essential N-terminal domain; IFD (also IFD^TRAP^) insertion in the fingers subdomain. **b** Interactions between the TRAP-wedge of TERT with the 5′ distal end of the DNA substrate and the 3′ template linker in the elongation state (top panel). The bottom panel shows the overlay of the same regions in the initiation (semi-transparent) and pre-termination (darker colours). **c**, **d** The effects of mutating the α1 helix and TRAP-wedge, respectively, on telomerase RAP. WT and mutant telomerase were characterised using immunoblotting, Northern blotting, and telomerase activity assay (see Source Data for uncropped blots and gels with size markers). For quantification, signals above the red line on the activity assay gel are defined as high RAP, whereas those below the red line are defined as low RAP. RC recovery control. **e**, **f** Quantification of relative processivity for WT and mutant samples shown in (**c**, **d**) respectively. Relative processivity is calculated by dividing the high RAP signal by the low RAP signal. Values were normalised to WT. Experiments were done in biological triplicate (see Source Data for replicate data). Data shown in e and f were quantified using a two-tailed *t* test. Data are presented as the mean values and error bars show the standard deviation. Significant *P* values are reported. Source data are provided as a Source Data file.

Multiple studies have implicated the TEN domain and IFD^TRAP^ of TERT in promoting telomerase processivity^13,23,59–68^. We previously identified residues in these two domains that form the so-called DNA anchor site^15^ (Fig. 4a). The anchor site stabilises the 5′ end of the DNA and prevents premature product dissociation during repeat synthesis^15^. In *Tetrahymena* telomerase, the binding of the IFD^TRAP^ to the 3′ region downstream of the RNA template has been suggested to prevent template backtracking, whereas the TEN domain did not interact with telomerase RNA^26^ (Supplementary Fig. 7f). In contrast, in all three of our structures, the human TEN domain and IFD^TRAP^ both interact with hTR (Supplementary Fig. 7e). How the hTR interaction with the TEN domain and the IFD^TRAP^ influences human telomerase activity and processivity remains unknown.

The TEN domain and IFD^TRAP^ form a wedge-like interface that splits the DNA–RNA duplex (Fig. 4a). Notably, we observed an extended β-hairpin structure within the IFD^TRAP^ (residues 747–757), which contacts both the DNA and RNA strands at the tip of the wedge (Fig. 4a, b). We refer to this region as “the TRAP-wedge”. One face of the wedge contains the DNA anchor site, which stably interacts with the 5′ end of the DNA substrate throughout the catalytic cycle (Fig. 4a, b). The other face, composed of the TRAP-wedge and a positively charged α-helix at the N-terminus of the TEN domain (α1: residues 8–17), contacts both the 3′ template linker and the adjacent P2a.1 stem in hTR (Fig. 4a). These observations are consistent with previous NMR studies showing that the yeast TEN domain binds single-stranded DNA and RNA emerging from the DNA–RNA duplex^69^. The contribution of the TEN domain to DNA binding also explains why in the absence of the TEN domain, protected DNA product lengths upon exonuclease treatment are shorter^35^.

From initiation to termination, the 3′ template linker and the 5′ DNA end undergo significant conformational changes, which are accommodated by corresponding shifts in the TEN domain and IFD^TRAP^ (Supplementary Fig. 9a–c). These rearrangements are more pronounced than those observed in the TERT-ring itself (Supplementary Fig. 9a). As the 3′ template linker becomes longer in the pre-termination state, the TRAP-wedge is pushed towards the DNA substrate, and the interactions between helix α1 of TERT and the 3′ template linker becomes destabilised (Fig. 4a).

To test the functional relevance of the TRAP-wedge, we reconstituted telomerase with mutations at residues H752 and H754 of this region, which wedge between the unpaired DNA and RNA away from the duplex, and performed activity assays (Fig. 4b, c). Interestingly, substituting H752 with alanine (H752A) resulted in a marked increase in both telomerase activity per TERT and RAP (Fig. 4c, lanes 1 and 2). A similar, though less pronounced, effect was observed with the H754A mutation (Fig. 4c, lane 3). The double H752A/H754A mutant displayed additive enhancement (Fig. 4c, lane 4). However, replacing H752 with tyrosine (H752Y) failed to enhance activity or processivity (Fig. 4c, lane 5), suggesting that the gain in function arises from removal of steric bulk at H752, rather than simple substitution. These results suggest that the TRAP-wedge may act as a steric barrier that modulates template translocation during RAP.

We next examined the role of α1 helix of TERT and its interaction with hTR in RAP (Fig. 4d). We mutated several arginine residues in this helix and the preceding loop, including R6, R8, R11 and R15, to either alanine or glutamate. With the exception of R6A and R8A, both charge reversal and alanine substitutions at other positions significantly impaired telomerase activity and RAP (Fig. 4d, lanes 2–9). These results highlight the importance of interactions between the TEN domain and both the 3′ template linker and the downstream P2a.1 stem in supporting repeat synthesis. Notably, the P2a.1 stem is specific to mammalian telomerase RNA^70^. The strong effect of this interaction on RAP may explain why the addition of the TEN domain in *trans* stimulates processivity in the human system more robustly than in *Tetrahymena*^71^. Taken together, the TEN domain and IFD^TRAP^ jointly regulate telomerase processivity by stabilising both the DNA substrate and critical RNA elements flanking the template.

## Discussion

**Fig. 5:**
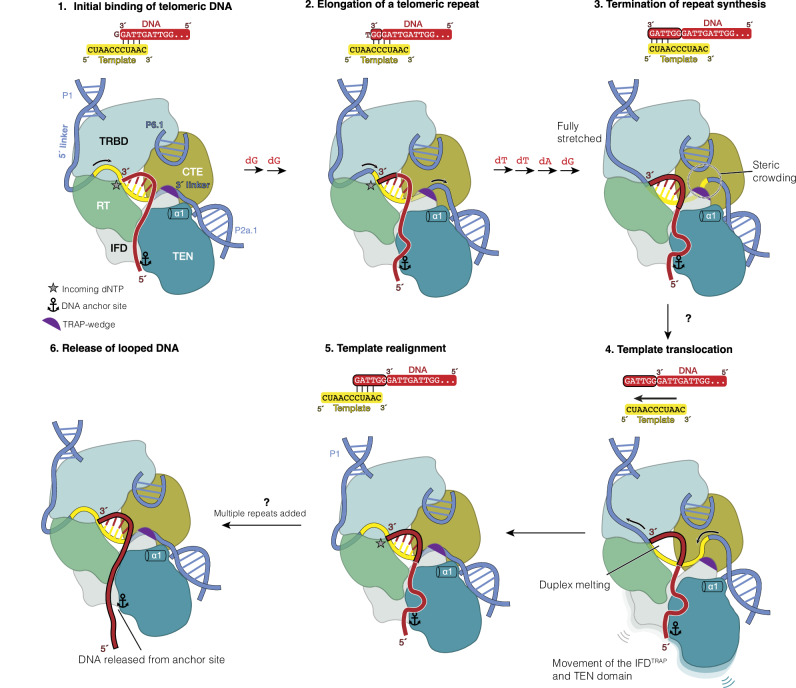
Model of telomeric repeat synthesis by human telomerase highlighting the roles of the TRAP wedge, α1 helix, and DNA anchor site of TERT. Repeat synthesis begins when the 3′ end of telomeric DNA base-pairs with the 3′ alignment region of the template, forming a four base-pair DNA–RNA duplex (step 1, initiation). In this state, the 3′ template linker is stabilised by interactions with the TRAP-wedge and the α1 helix of TERT. Upon incorporation of the incoming nucleotide, the duplex extends to five base-pairs. During elongation (step 2), six nucleotides are sequentially added to the DNA 3′ end. Each nucleotide addition is coupled with melting of one base pair at the distal end, maintaining a constant four base-pair duplex. Once the 5′ end of the template is reached, the 5′ template linker is fully stretched, halting repeat synthesis (step 3, termination). In contrast, the ssRNA is extruded from the 3′ template linker, causing steric strain and disrupting its interaction with the TRAP-wedge and α1 helix. These tensions lead to duplex melting, but the DNA remains bound via interactions with the TERT-ring and the DNA anchor site (step 4, translocation). To relieve the strain, the RNA template retracts through the active site and re-aligns with the DNA via base-pairing, re-establishing the initiation state (step 5, realignment). The 5′ end of the newly synthesised DNA loops out and is eventually released from the anchor site after multiple rounds of repeat synthesis (step 6).

In this study, we biochemically and structurally captured the catalytic core of human telomerase stalled at three distinct states of its RAP catalytic cycle. Our findings corroborate earlier biochemical data and define key molecular interactions that underlie different steps of RAP. These insights allow us to propose a detailed model of telomeric repeat synthesis by the human enzyme highlighting the involvement of the DNA anchor site, the TRAP-wedge and the α1 helix of TERT in this process (Fig. 5).

Telomeric repeat synthesis initiates when the 3′ end of the telomeric DNA substrate forms a 4 base-pair duplex with the alignment region of the RNA template (Fig. 5, step 1). Conserved motifs within the TERT-ring stabilise this 4 base-pair duplex (Fig. 2a-g), while the unpaired distal ends of the DNA–RNA hybrid are engaged by the TEN domain and IFD^TRAP^ (Fig. 4a). Notably, we identify two previously uncharacterised features – TRAP-wedge and helix α1 of the TEN domain – that interact with the 3′ template linker, downstream of the templating region.

During the elongation phase, six nucleotides are added to the DNA using the templating sequence of hTR (Fig. 5, step 2). Each incoming nucleotide base-pairs with an RNA base in the templated region of the RNA template, transiently extending the duplex to 5 base pairs. After each addition, the template advances by one base into the active site, while the 3′ end of the DNA shifts outward. The most distal DNA–RNA base-pair is melted by the “zipper head” L981^24^, resetting the duplex to 4 base pairs.

As the template moves through the active site, the 5′ template linker shortens and the 3′ template linker loops out (Fig. 5, steps 2 and 3). Upon reaching the termination state (Fig. 5, step 3), our structures suggest that duplex dissociation and template translocation are cooperatively facilitated by several factors: tension in the fully stretched 5′ linker, weakened DNA–RNA base-pairing, steric hindrance downstream of the template, and reduced interactions between helix α1 of TERT and the 3′ template linker (Fig. 5, step 3).

DNA protection assays revealed that the length of protected DNA increases progressively from the initiation to termination states^35^. Since the duplex length stays constant, our structures and these data suggest that the DNA remains anchored while looping out between the anchor site and the end of the duplex. This looped DNA may represent additional steric strain against the bulged 3′ template linker (Fig. 5, step 3). Supporting this model, optical tweezer studies show that telomerase can release multiple repeats in a single burst during RAP, indicating that anchor site–DNA contacts persist across multiple rounds of repeat synthesis^72^. Additional single-molecule studies further suggest that the looped-out DNA may fold into G-quadruplexes^73^. Recent evidence also implicates replication protein A (RPA) as a telomerase processivity factor^74^. It would be of interest for future studies to address how the 5′ end of the telomeric DNA is coordinated between telomerase, TPP1–POT1 and RPA.

Although we did not capture the translocation state, previous studies have proposed a single-stranded DNA retention surface that holds DNA after duplex melting and during template translocation^5,34^. Based on our structural data (Fig. 2b, d, f), we propose that this surface comprises several TERT elements, including motifs E, T, and 3, as well as the thumb helix and thumb loop (Fig. 5, step 4). This DNA retention surface works in concert with the anchor site in the TEN domain and IFD^TRAP^ to retain DNA during translocation (Fig. 4a).

Concurrently, the displaced RNA template rethreads through the TERT active site and is re-engaged by base-pairing with the DNA product (Fig. 5, step 5). The TRAP-wedge and helix α1 may facilitate this re-engagement by re-forming interactions with the 3′ template linker, as observed in the initiation state (Fig. 4a). Across structures of human and *Tetrahymena* telomerases, including those presented here, the TEN domain and IFD^TRAP^ display greater conformational flexibility than the TERT-ring^7,26^. This mobility may accommodate template movement during translocation (Fig. 5, step 4). However, since the TEN domain and IFD^TRAP^ also anchor the DNA, their movement relative to the TERT-ring must be tightly regulated to prevent DNA release. Supporting this, mutation of Y667 in motif 3a of TERT, which interacts with IFD^TRAP^, results in reduced telomerase activity and RAP^34,38^ (Supplementary Fig. 9a, inset). To fully elucidate the mechanism of translocation, future work will require biochemical strategies to kinetically trap this elusive state for structural characterisation.

In conclusion, our work reveals how TERT and hTR coordinate telomeric DNA during RAP and provide an invaluable foundation for further exploration of RAP regulation by telomerase cofactors and for the development of therapeutic strategies targeting telomerase processivity in cancer.

## Methods

### Telomerase reconstitution and purification

Human telomerase was expressed in Expi293F^TM^ cells (Thermofisher, Cat# A14527) by co-transfecting pcDNA3.1-ZZ-TEV-twin-Strep-SUMO*-TERT and pcDNA3.1-U3-hTR-HDV^6,8,33^. TEV is Tobacco Etch Virus protease cleavage site, SUMO* is SUMOstar protease cleavage site (LifeSensors, Cat# SP4110) and HDV is the hepatitis delta virus ribozyme. Cells were grown in Expi293F^TM^ expression medium (Gibco, Cat# A1435101) and transfected at a density of ~2 × 10^6^ cells/mL. A total of 1.1 μg DNA per mL cell culture was transfected with a TERT/hTR DNA ratio of 1:4 using homemade polyethylenimine (PEI) at a final concentration of 0.003 mg/mL to enhance transfection. Transfected cells were cultured at 37 °C for ~48 h and then harvested by centrifugation. Cell pellets were resuspended in hypotonic lysis buffer (HLB) [20 mM HEPES pH 8.0, 2 mM MgCl_2_, 0.2 mM EGTA, 10% v/v glycerol, 1 mM DTT, 0.2 mM PMSF, 1:200 v/v protease inhibitor cocktail (Sigma, Cat# P8340)] and lysed by three freeze-thaw cycle. Lysates were then supplemented with NaCl (300 mM), centrifuged (21,300 g, 15 min), and diluted in a 1:1 v/v ratio with HLB supplemented with 0.2% v/v IGEPAL CA-630. Lysates were snap frozen in liquid nitrogen and stored at −70 °C until used.

### Reconstitution of human telomerase with DNA and TPP1–POT1–TIN2 (TPT)

Streptavidin agarose resin (Thermo Scientific, Cat# 20361) was bound by 10 μM 5′ biotinylated 2′-O-methyl oligonucleotide (IDT, sequence: 5′-bio- CTAGACCTGTCATCAmGmUmUmAmGmGmGmUmUmAmG, where m denotes 2′-O-methyl and bio denotes biotin adduct) for 1 h at room temperature. Excess oligonucleotide was washed away with wash buffer (20 mM HEPES NaOH pH 8.0, 150 mM NaCl, 2 mM MgCl_2_, 0.2 mM EGTA, 10% glycerol, 0.1% IGEPAL CA-630, 1 mM DTT and 0.2 mM PMSF).

Telomerase lysates were added to pre-equilibrated streptavidin agarose resin (Thermo Scientific, Cat# 20361), prebound to a 5′ biotinylated 2′-O-methyl oligonucleotide for 2.5–3 h at room temperature. The resin was washed with wash buffer and eluted with a competitor oligonucleotide (IDT, sequence: CTAACCCTAACTGATGACAGGTCTAGddC where ddC is dideoxycytosine). The eluate was next incubated with MagStrepXT resin (IBA Life Sciences, Cat# 2-4090-002) for 2 h at 4 °C. After washing three times with wash buffer, the sample was incubated with 10 μM DNA primer [(TTAGGG)_5_TTAG for initiation, (TTAGGG)_5_ for elongation, or (TTAGGG)_5_TTA for pre-termination] and 100 μM of a non-hydrolysable analogue of the incoming nucleotide [dGpCpp for initiation and pre-termination complexes (Jena Bioscience, Cat# NU-431S) or dTpCpp for elongation (Jena Bioscience, Cat# NU-895S)] for 45 min at room temperature. All telomeric DNA primers used were synthesized by IDT. After washing, the purified shelterin sub-complex TPP1–POT1–TIN2 (TPT) was incubated with telomerase at a final concentration of 0.15 mg/ml. TPT was expressed in *S. Frugiperda* (Sf9) (Oxford Expression Technologies Ltd, Cat# 600100) and purified as previously described^15^. TPT was incubated for 1 h at 4 °C. The final sample was eluted by overnight cleavage with sumostar protease (LifeSensors, Cat# SP4110) at 4 °C. Fractions were analysed by SDS-PAGE and silver staining (Invitrogen, Cat# LC6070).

### Cloning of TERT and hTR mutants

Mutagenesis primers were designed using NEBaseChanger and synthesized commercially by IDT (Supplementary Data 5). Site-directed mutagenesis was performed on pcDNA3.1-ZZ-TEV-twin-Strep-SUMO*-TERT and pcDNA3.1-U3-hTR-HDV constructs using the Q5 Site-Directed Mutagenesis Kit (NEB, Cat# E0554). All mutations were verified by DNA sequencing. For downstream transfections, plasmid DNA was prepared using the PureLink HiPure Midiprep Kit (Invitrogen, Cat# K210005).

### Telomerase activity assays

Telomerase primer extension assays were conducted in a reaction buffer containing 50 mM Tris-acetate pH 8.0, 50 mM potassium acetate, 3 mM MgCl₂, 1 mM EGTA, 1 mM spermidine, 5 mM β-mercaptoethanol, 5 mM DTT, 250 μM dTTP, 250 μM dATP, 5 μM unlabeled dGTP, and 0.1 μM α-³²P-dGTP (3,000 Ci mmol⁻¹, 10 mCi ml⁻¹; Hartmann Analytic GmbH, Cat# FP204). Reactions also included 500 nM (TTAGGG)₅ primer and 5 mM DTT, and were incubated at 30 °C for 45 min. To terminate the reaction, stop buffer (50 mM Tris-HCl pH 7.5, 20 mM EDTA, and 0.2% SDS) was added. Nucleic acids were extracted using phenol:chloroform:isoamyl alcohol (Thermo Scientific, Cat# 17908), and precipitated with ethanol in the presence of a ³²P-labeled 24nucleotide-long DNA as a recovery control (RC).

DNA extension products were resolved on a 10.5% denaturing polyacrylamide (19:1 acrylamide:bisacrylamide) Tris-borate-EDTA (TBE) gel, run at 500 V for 2 h and 15 min. The gel was dried at 80 °C for 45 min and exposed to a phosphor screen overnight. Imaging was performed using an Amersham Typhoon Biomolecular Imager (Cytiva). Quantification of three independent biological replicates was done using ImageJ.

Total telomerase activity was calculated by dividing the activity in each WT or mutant lane by the RC, yielding a relative activity value (Activity/RC). This value was then corrected for differences in TERT expression levels by dividing the activity by the corresponding TERT/tubulin ratio from the lysate. This was then normalised to the WT value. Relative processivity was determined by calculating the telomerase activity corresponding to products with > 10 repeats by the RC (Activity > 10/RC). To account for the expression differences, this value was adjusted by dividing it by the corresponding TERT/tubulin ratio from the lysate, and subsequently normalised to the WT. Processivity ratio (High RAP/Low RAP) was calculated by dividing the telomerase activity corresponding to products with > 10 repeats by that from products with < 10 repeats. This ratio was then corrected for differences in TERT expression by dividing it by the TERT/tubulin ratio in the lysate and normalised to 1 in the WT condition. A two-tailed t-test was performed in Prism and significant *P*-values are reported.

### Immunoblotting

Protein extracts containing telomerase were resolved using a 4–12% Bis-Tris NuPAGE gel (Invitrogen, Cat# NP0321BOX) followed by transfer to a nitrocellulose membrane. Membranes were blocked for a minimum of 2 h at room temperature in 5% non-fat dry milk dissolved in phosphate-buffered saline (PBS) with 0.1% Tween-20 (PBST), and then incubated overnight at 4 °C with primary antibodies: mouse anti-Alpha tubulin (1:20000; Proteintech, Cat# 66031-1-Ig, lot 10004185) and rabbit anti-TCAB1 (1:1000; Proteintech, Cat# 14761-1-AP). After three PBST washes, membranes were probed with secondary antibodies (1:5000 dilution): either goat anti-Rabbit Alexa Fluor 680 (Abcam, Cat# ab175773, lot GR222353-8) or goat anti-Mouse Alexa Fluor 680 (Abcam, Cat# ab175775, lot GR3273649-2). Following final washes, blots were imaged using a LI-COR Odyssey imaging system. Signal intensities were quantified using ImageJ.

### Northern blotting

Total RNA was purified from crude lysates of cells co-expressing TERT and hTR WT and mutants constructs using TRIzol reagent (Invitrogen, Cat# AM9738). Purified RNA samples were resolved on a 5% denaturing polyacrylamide gel (19:1 acrylamide:bis-acrylamide) containing 7 M urea in 0.6x TBE buffer. Following electrophoresis, the RNA was transferred to a Hybond-N⁺ nylon membrane (Cytiva, Cat# RPN303) and cross-linked using a Stratalinker UV crosslinker for 2 min. The membrane was pre-incubated with Church’s buffer [0.5 M phosphate buffer, pH 7.2; 1% bovine serum albumin (BSA); 1 mM EDTA; and 7% SDS] supplemented with 15% formamide for 30 min at 50 °C. Hybridisation was carried out overnight at 50 °C using ³²P-end-labelled DNA probes specific for the CR4/5 (IDT, sequence: TCG CGG TGG CAG TGG GTG CCT C) and PK/t (IDT, sequence: TAG AAT GAA CGG TGG AAG GCG G) domains of hTR. A probe against 7SL RNA (IDT, sequence: TGCTCCGTTTCCGACCTGGGCCGGTTCACCCCTCCTT) was used as a loading control. After hybridisation, the membrane was washed twice with 4x SSC containing 0.1% SDS, followed by two washes with 2x SSC and 0.1% SDS, each for 20 min. The membrane was then air-dried and exposed to a phosphor screen overnight. Signal detection was performed using an Amersham Typhoon Biomolecular Imager (Cytiva).

### Cryo-EM sample preparation and data collection

Purified telomerase complex was crosslinked with 0.5 mM bis(sulfosuccinimidyl)suberate (BS3) (Thermo Fisher, Cat# A39266) for 1 h on ice. Crosslinking was quenched with quench buffer (200 mM Tris pH 8.0, 150 mM NaCl, 2 mM MgCl_2_, 0.05% IGEPAL CA-630, and 1 mM DTT). The sample was dialysed for 2 h into cryo-EM buffer (20 mM HEPES NaOH pH 8.0, 150 mM NaCl, 2 mM MgCl_2_, 0.05% IGEPAL CA-630, 1% trehalose, 1 mM DTT). C-flat 4/2-4Cu T50 grids (Protochips, Cat# 71150) coated in continuous layer of carbon support (~5–6 nm homemade carbon) were discharged for ~10 seconds with a sputter coater glow discharger (model Edwards S150B). Grids were prepared using the Vitrobot system (Thermo Fisher, Mark IV). Sample (3 μL) was applied to the grid, incubated for 2 min at 4 °C and 100% humidity and blotted with filter paper (Whatman) for 4.5–6 s before plunge freezing in liquid ethane.

Cryo-EM data were collected on the Titan Krios (300 keV) transmission electron microscope (Thermo Fisher) with Gatan K3 direct electron detector and GIF Quantum energy filter. Data were collected at 81000x nominal magnification (physical pixel size of 1.059 Å) with a 20 eV slit width and defocus range of −1 to −2.5 μm. An approximate dose of 45–50 e/Å^2^ across an exposure time of 2.5–3.0 s was used, and micrographs were fractionated to give an approximate electron dose of 1 e/Å^2^/frame (Supplementary Fig. 3a–c). A total of 186,852 micrographs were collected for the three complexes [61,257 for the initiation complex (Supplementary Fig. 4a), 56,415 for the elongation complex (Supplementary Fig. 4b), and 69,180 for the pre-termination complex (Supplementary Fig. 4c)].

### Cryo-EM data processing

All data processing was performed in RELION5.0^75,76^ unless stated otherwise. Movie frames were motion-corrected and dose-weighted using RELION’s own implementation and CTF was estimated using CTFFIND-4.1 in RELION^77^. Each complex was collected in multiple datasets and processed as described below. Of note, some of the data presented here were also used to resolve a dimeric state of telomerase as described in another study^28^.

#### Telomerase initiation complex

We collected 61,257 micrographs for this complex across 2 datasets. Initially, both datasets were processed separately in the same way (Supplementary Fig. 4a). Particles were picked using reference-based auto-picking. Unbinned 2D classes from previous cryo-EM data were used as references^15^. Particles were extracted at bin 8 with a box size of 56^2^ pixels and then subject to initial 3D classification with angular sampling of 7.5° and a regularisation parameter *T* of 4 for 50 iterations. Classes that show good telomerase features were selected and subjected to 2D classification using the EM algorithm in RELION. Particles from good 2D classes were selected, combined and unbinned for an initial 3D refinement. This refinement yielded medium-resolution reconstructions (7.7 Å for dataset 1, and 10.8 Å for dataset 2).

At this stage, particles from dataset 1 were polished by Bayesian polishing and re-refined. To improve the resolution of the catalytic core, signal subtraction was performed on the catalytic core lobe of the map. Particles were recentered, and the box size was reduced to 280^2^ pixels to speed up computation. 3D classification was carried out on the signal subtracted particles with angular sampling of 7.5° and regularisation parameter *T* of 4 for 50 iterations. One class clearly showed high-resolution features and was taken forward for further data processing (248,966 particles).

After initial refinement of whole telomerase particles, dataset 2 underwent the same signal subtraction, recentering, and re-boxing procedure as dataset 1. 3D classification of these particles was performed in two steps. First, 3D classification of 50 iterations with angular sampling of 7.5° and a regularisation parameter *T* of 4 was performed. Then, the angular sampling was reduced to 3.5 degrees for another 10 iterations to improve classification. Again, a dominant class with high resolution features was isolated and taken forward. The particles from this class were polished by Bayesian polishing (240,937 particles) and subsequently combined with the best signal subtracted particles from dataset 1.

The combined particles from datasets 1 and 2 (489,903 particles) were refined together to yield a 3.7-Å map. To improve the quality of the map around the active site, a circular local mask was used to remove any density outside of the masked region. Using this mask, alignment free 3D classification was performed. Various *T* values were tested, and the most optimal *T* value was selected (*T* = 40). The class showing the strongest density for DNA and dNTP in the active site was picked and subject to 3D refinement, giving a map at 3.5-Å resolution after post-processing (Supplementary Fig. 5a–d).

#### Telomerase elongation complex

56,415 micrographs were collected over 2 datasets. Initially, both datasets were processed separately in the same way (Supplementary Fig. 4b). A Topaz-trained model was used for particle picking^78^, followed by extraction of particles with a figure-of-merit (FoM) of −3.5 or higher. Particles were extracted at bin 8 with a box size of 56^2^ pixels. Initial 3D classification was carried out in two steps using a regularisation parameter *T* of 4. The first 25 iterations were performed with angular sampling of 15°. The angular sampling was then reduced to 7.5° for another 25 iterations. Promising classes were selected for 2D classification using the EM algorithm. The best 2D classes were selected, combined, unbinned, and subjected to another round of 2D classification with the same parameters to remove any remaining junk particles.

For dataset 1, the selected particles (558,049 particles) went through an initial 3D refinement, resulting in a low-resolution map (16.9 Å). Signal subtraction was used to focus on the catalytic core. Particles were recentered, and the box size was decreased to 280^2^ pixels. 3D classification of the catalytic core was performed with angular sampling of 7.5°, and a regularisation parameter *T* of 4 for 50 iterations. This yielded a good subset of particles (186,403 particles) to be carried forward for further processing.

The original un-subtracted subset of particles from dataset 1 was joined with those selected from 2D classification of dataset 2 (1,290,512 particles). The joined particle stack was refined to 10.8-Å resolution. Particles were then polished by Bayesian polishing and re-refined to 10.5-Å resolution. Signal subtraction was performed on the catalytic core, followed by recentering, and reduction of the box size to 280^2^ pixels. 3D classification on the catalytic core was performed initially with angular sampling of 15° for the first 25 iterations, then the angular sampling was reduced to 7.5° for a further 25 iterations. A regularisation parameter *T* value of 4 was used for all iterations. This classification separated further junk particles and yielded the best class of 935,853 particles. We refined these particles to 3.2-Å resolution with Blush regularisation^79^. This map was used for further focussed classification. First, a small spherical mask was used around the telomerase active site for 3D classification without alignment for 50 iterations with a regularisation parameter *T* value of 1200. We isolated a subset of 668,791 particles with dTpCpp bound. These particles were refined without Blush regularisation to 3.5-Å resolution. Another local mask around the TEN domain and TPP1 part of the map was created using the molmap function in ChimeraX^80^. Further alignment-free 3D classification with this mask removed any particles without TPP1 bound. A final subset of particles (202,390 particles) was refined to 3.6 Å. Particles were then subjected to further CTF refinement (beam tilt, anisotropic magnification, and defocus)^81^. Re-refinement of these particles resulted in an improved map of 3.2 Å after post-processing (Supplementary Fig. 5f–i).

#### Telomerase pre-termination complex

Three independent datasets (69,180 micrographs) were collected and processed separately but in the same manner (Supplementary Fig. 4c). Particles were picked through optimised reference-based auto picking. Particles were extracted at bin 8 with a box size of 56^2^ pixels. Extracted particles underwent 3D classification for 50 iterations with angular sampling of 7.5° and regularisation parameter *T* of 4. Classes with good features were taken forward to 2D classification with the EM algorithm. Good 2D classes were selected, combined, and unbinned before 3D refinement. Initial 3D refinement yielded maps of 13–14 Å resolution for each of the datasets.

Following 3D refinement, signal subtraction was performed on the telomerase catalytic core. Particles were recentered, and the box size was reduced to 280^2^ pixels. 3D classification of the catalytic core was carried out in two steps. An initial classification with angular sampling of 7.5° and a regularisation parameter *T* of 4 was done for 25 iterations for dataset 1, and 50 iterations for datasets 2 and 3. The angular sampling was then decreased to 3.7°, for further iterations, until the particle distribution across the classes were stabilised. The most featureful classes were selected. Particles were reverted to their original un-subtracted particles for Bayesian polishing. Polished particles were then combined into one single stack of 896,466 particles and subjected to a further round of 3D classification to remove any remaining junk particles. The first 25 iterations of the classification were performed with 7.5° angular sampling, a regularisation parameter *T* of 4, followed by a further 25 iterations with 3.7° angular sampling. Two classes of 505,879 particles with high-resolution features were selected and forwarded to 3D refinement. A reconstruction of the catalytic core was obtained at 3.7-Å resolution. A spherical mask including the active site duplex and the flexible 5′ and 3′ linkers flanking the template region in hTR was used for focussed 3D classification without alignment. We tested different regularisation parameter *T* values, and the optimal 3D classification used the standard *T* value of 4. The class with the most complete occupancy of the active site and strongest density for the RNA linkers (95,317 particles) was selected and subject to 3D classification. A final map with a resolution of 3.8 Å after post-processing (Supplementary Fig. 5k–n).

#### Map refinement and validation

For all reported maps, refinements were carried with independent half datasets. Resolution of refinements are reported based on the gold standard Fourier Shell Correlation (FSC) = 0.143^82^ (Supplementary Fig. 5a, f, k). FSCs were calculated through post-processing of maps with a soft mask, corrected for the Modulation Transfer Function (MTF) of the detector, and B-factor sharpening. B-factors were either calculated by RELION, or user-defined based on the quality of the map. Directional FSC was calculated for all maps using the 3D FSC server, (https://3dfsc.salk.edu/)^83^ (Supplementary Fig. 5b, g, l). 2D histograms of Euler angles were created using scripts from; (https://githubhelp.com/Guillawme/angdist) (Supplementary Fig. 5c, h, m). Map local resolution was calculated in RELION (Supplementary Fig. 5d, i, n).

### Model building and refinement

The three models were built using the same methodology described here. First, a previous model of the telomerase catalytic core with DNA and TPP1 bound (PDB 7QXA)^15^ were docked into the post-processed map in ChimeraX^80^. The model was allowed to adjust to fit into the map better using ISOLDE^84^. Torsion restraints were initially added to the TEN domain and TPP1 as the density in these regions of the map is lower resolution. Distance restraints were also added to hTR to prevent melting of secondary structures in regions of poor density. For challenging regions, a blurred map was generated to aid model building. Rotamer and Ramachandran outliers were fixed in ISOLDE, and all residues were assessed for their conformation and fit into the map. For the different complexes, the DNA, and RNA linkers and template regions of hTR (nucleotides 38–63) were rebuilt manually in COOT^85^ to fit with the DNA substrate added and hTR conformational changes. Non-hydrolysable nucleotide ligands were added in COOT and fit into the density as best as possible. For the initiation and pre-termination complexes, the non-hydrolysable analogue of dGTP, dGpCpp (ligand code: 1GC) was added. For the elongation complex, a non-hydrolysable analogue of dTTP (dTpCpp ligand code: A1ACD). After manual rebuilding of the DNA and hTR, we use ISOLDE^84^ to improve the model fit to the map, and optimise the clashes and geometry where possible.

Following model building, model refinement by Phenix real-space refinement^86^ was performed using restraints generated from ISOLDE^84^. AceDRG^87^ was used to generate an mmCIF file for the ligand. To further improve model-to-map fit, models refined in Phenix were further refined in Servalcat^88^ using protein secondary restraints generated with PROSMART^89^ and nucleic acid restraints produced by LIBG^90^ (Supplementary Fig. 5e, j, o).

Models were analysed with Molprobity^91^ to assess the geometries of the final models (Supplementary Table 1). Model-versus-map FSC calculations were carried out in Phenix^86^ (Supplementary Fig. 5a, f, k). Maps and models were visualised in ChimeraX^80^ and Pymol (www.pymol.org). Figures were created with ChimeraX^80^, Pymol (www.pymol.org) and Adobe Illustrator.

### DRRAFTER modelling

For each of the three cryo-EM maps, an ensemble of models for the RNA-DNA hybrid at the active site was modeled using DRRAFTER^54^ using the unsharpened map, which has more uniform density for these regions. The DNA strand up to the most 3′ end (residue 34 for initiation, 30 for elongation and 33 for pre-termination) and RNA residues 38 to 63 were modeled, with the remainder of the model fixed. The five base-pairs between the RNA and DNA/the incoming nucleotide were enforced (initiation: DNA 31–34 and RNA 51–55; elongation: DNA 27–30 and RNA 49–53; pre-termination: DNA 30–33 and RNA 46–50), no other constraints were used. DRRAFTER currently remodels only RNA residues; to enable sufficient sampling of strand conformation, the DNA strand was modeled as RNA in DRRAFTER and then converted back to DNA. At least 20,000 models were sampled for each cryo-EM map, and the top 10 models were used to represent the ensemble (Supplementary Fig. 8d–f, Supplementary Data 2–4).

### Reporting summary

Further information on research design is available in the Nature Portfolio Reporting Summary linked to this article.

## Supplementary information

Supplementary Information

Description of Additional Supplementary Files

Supplementary Data 1

Supplementary Data 2

Supplementary Data 3

Supplementary Data 4

Supplementary Data 5

Reporting Summary

Transparent Peer Review file

## Source data

Source data

## Data Availability

Cryo-EM maps of the catalytic core of telomerase bound to TPP1 in the initiation, elongation and pre-termination states generated in this study have been deposited in the Electron Microscopy Data Bank under accession codes EMD-54920, EMD-54921 and EMD-54922, respectively. Refined atomic coordinates for the catalytic core at the initiation, elongation and pre-termination states have been deposited in the Protein Data Bank under accession codes 9SHY, 9SHZ and 9SI0, respectively. A summary of published TERT mutagenesis data relevant for this work is provided as Supplementary Data 1. Coordinates of hTR from DRRAFTER modeling generated in this study are provided as Pymol sessions in Supplementary Data 2 to 4. Sequences of DNA primers used for mutagenesis experiments are provided in Supplementary Data 5. Materials are available under a material transfer agreement with the MRC Laboratory of Molecular Biology from the corresponding author. Requests for the Pymol script for plotting conformational change should be addressed to the corresponding author. Source data are provided with this paper.
