# Supplementary material for: Structures of nucleotide-bound human telomerase at several steps of its telomeric DNA repeat addition cycle
Source: Nat Commun. 2026 Jan 21;17:1847. doi: 10.1038/s41467-026-68560-8 (PMC12920920; doi:10.1038/s41467-026-68560-8)
Supplement: Supplementary file 1 — Supplementary Information [file 41467_2026_68560_MOESM1_ESM.pdf]

SUPPLEMENTARY INFORMATION

Structures of nucleotide-bound human telomerase at several steps of its telomeric DNA repeat addition cycle

Sebastian Balch[1], Elsa Franco-Echevarría[1], George Ghanim[1,6], Rachael C Kretsch[2], Rhiju Das[3,4,5], Thi Hoang Duong Nguyen[1]*

[1] Medical Research Council Laboratory of Molecular Biology, Francis Crick Avenue, Cambridge, CB2 0QH, United Kingdom
[2] Biophysics Program, Stanford University, Stanford, California 94305, USA
[3] Department of Biochemistry, Stanford University, Stanford, California 94305, USA
[4] Department of Physics, Stanford University, Stanford, California 94305, USA
[5] Howard Hughes Medical Institute, Stanford, California 94305, USA
[6] Present address: Department of Molecular Biology, Princeton University, Princeton, NJ, USA.
*Corresponding author: knguyen@mrc-lmb.cam.ac.uk

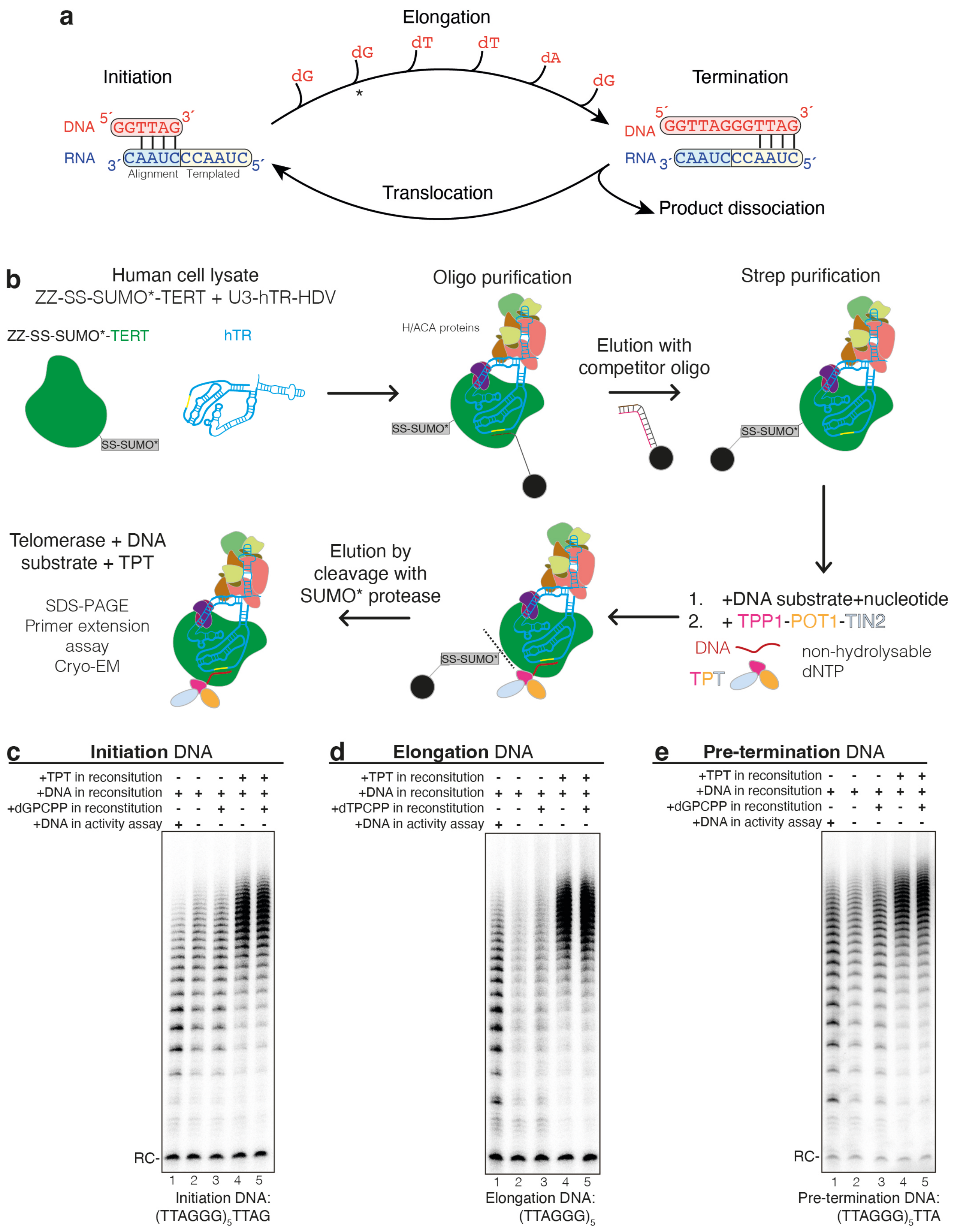

Supplementary Fig. 1 | Biochemical reconstitution and purification of telomerase in different catalytic states with the shelterin sub-complex TPP1–POT–TIN2.

a, Schematic of the RAP cycle of human telomerase. Previous structures of human telomerase were captured with TTAGGG permutation[1-3], corresponding to the elongation state as indicated by the asterisk in the RAP cycle. **b,** Expression, purification, and assembly of human telomerase with DNA and TPP1–POT1–TIN2 for cryo-EM studies. **c–e,** Telomerase activity assays showing the effects of the incoming nucleotide and TPT on the retention of the DNA substrate corresponding to the initiation **(c)**, elongation **(d)**, and pre-termination **(e)** states of the RAP cycle during reconstitution with telomerase. Source data are provided as a Source Data file.

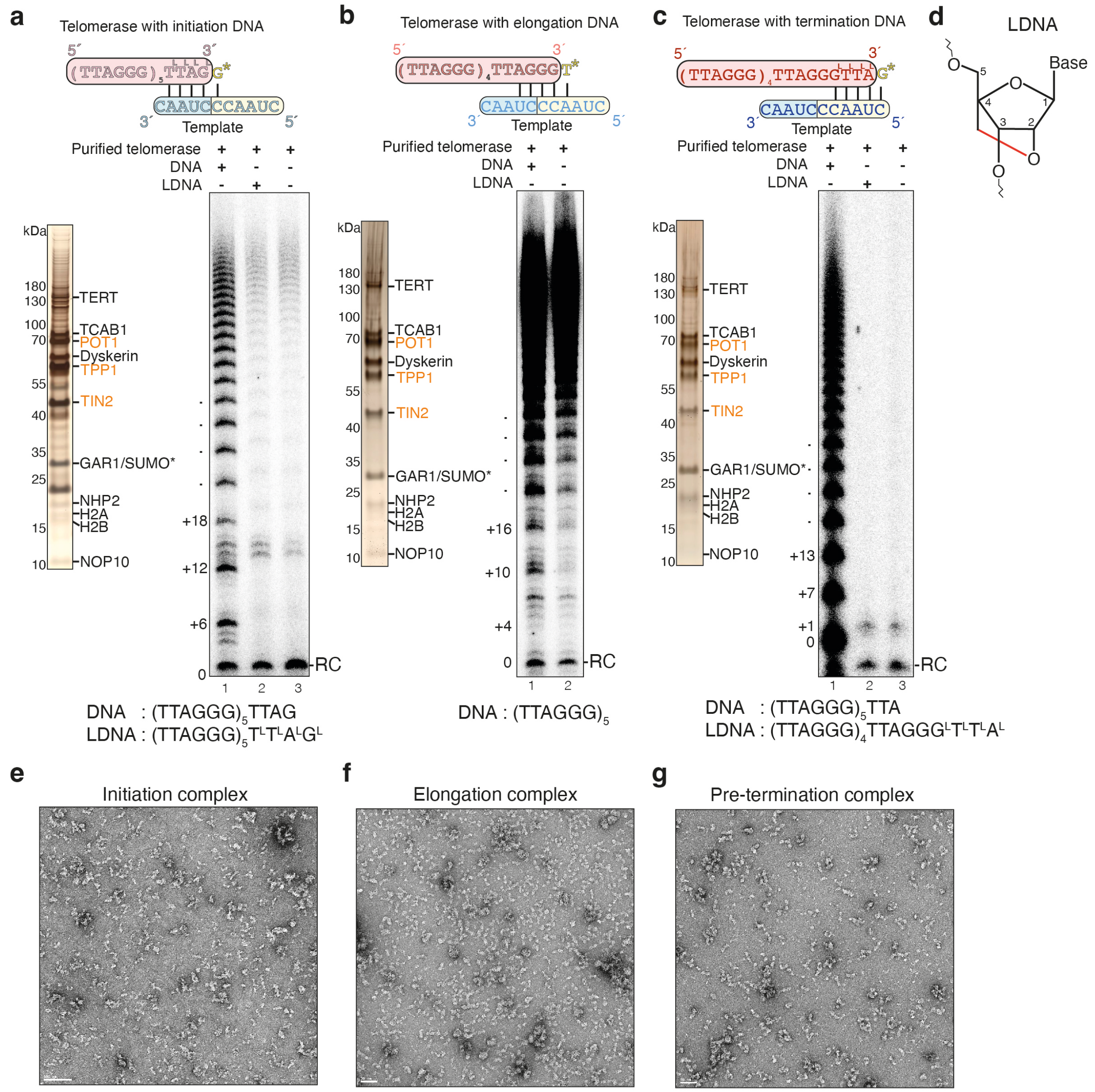

Supplementary Fig. 2 | Validation of purified complexes of telomerase with TPT, DNA and incoming nucleotide at the initiation, elongation and pre-termination states of the RAP cycle. a–c, Silver-stained SDS-PAGE and telomerase activity assay validation for the initiation **(a)**, elongation **(b)**, and pre-termination complexes **(c)**. RC; recovery control. **d,** Chemical structure of the sugar moiety in a locked nucleotide. Telomerase was purified in the presence of the corresponding DNA substrate, as shown in the top schematic (Supplementary Fig. 1b). For the initiation and pre-termination states, we used DNA substrates with the final four 3′ nucleotides chemically modified with locked nucleotides (LDNA). We then assayed the purified telomerase in the absence of additional telomeric DNA to verify whether the DNA was bound to the enzyme

(panel **a**, lane 3; panel **b**, lane 2; panel **c**, lane 3). As positive controls, we also assayed the same purified telomerase in the presence of additional telomeric DNA, both non-modified and LDNA, to ensure that telomerase is active. RC, recovery control. **e–g,** Representative negative stain electron micrographs of the telomerase initiation **(e)**, elongation **(f)**, and pre-termination **(g)** complexes. These negative stain EM experiments were performed once to examine the quality of the purified telomerase complexes. Samples with good quality were subsequently subjected to cryo-EM sample preparation. Therefore, no repeat of the negative stain EM experiments was required. Source data are provided as a Source Data file.

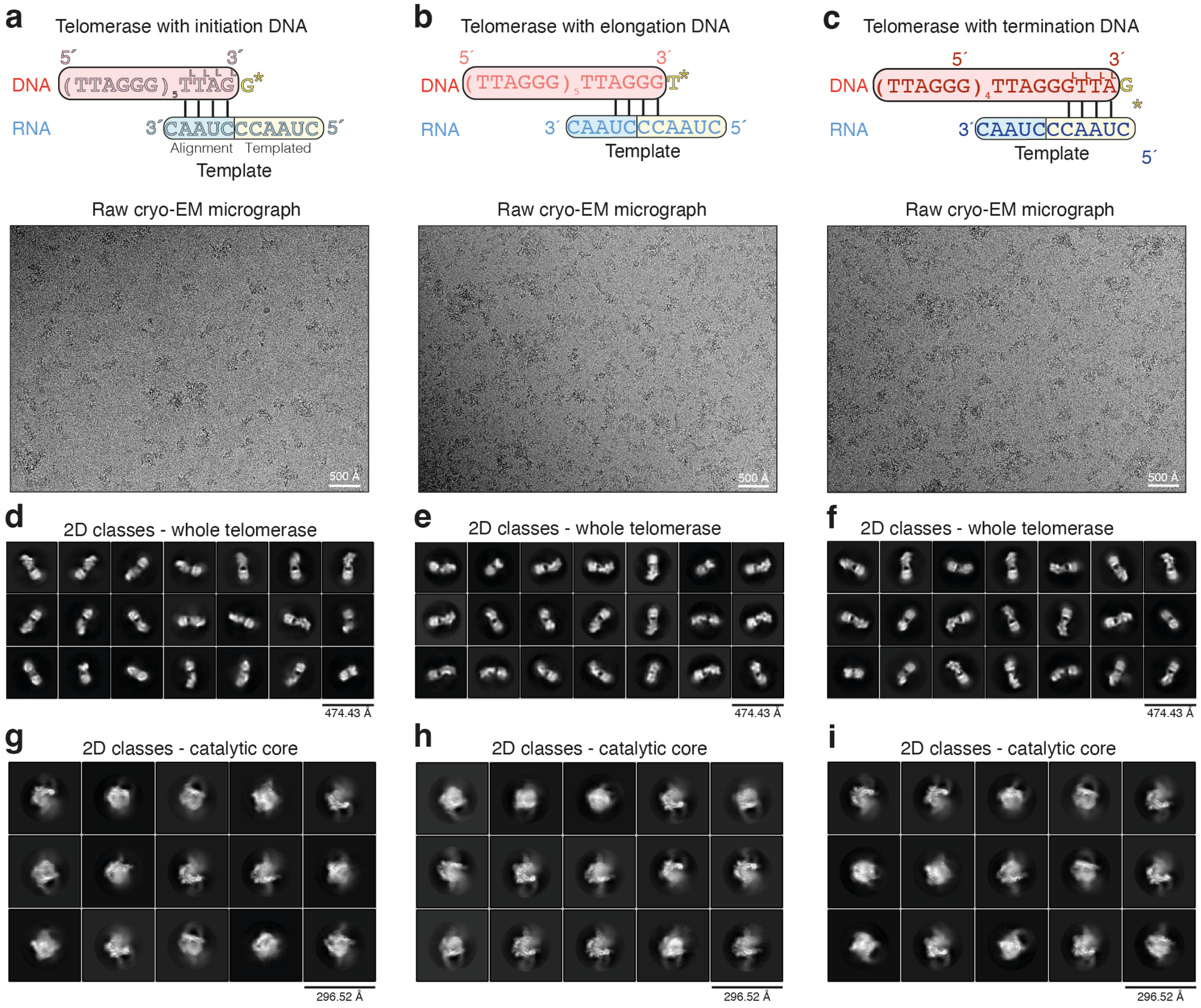

Supplementary Fig. 3 | Cryo-EM micrographs and 2D class averages for the telomerase complexes reconstituted.

a–c, Representative cryo-electron micrographs with schematics of the DNA-RNA duplex in the states being captured for the initiation **(a)**, elongation **(b)**, and pre-termination **(c)** complexes. **d–f,** Representative 2D class averages of the whole telomerase enzyme for the initiation **(d)**, elongation **(e)**, and pre-termination **(f)** complexes. **g–i,** Representative 2D class averages of the telomerase catalytic core after signal subtraction for the initiation **(g)**, elongation **(h)**, and pre-termination **(i)** complexes. These cryo-EM experiments were performed multiple times (n>3) to obtain the optimal cryo-EM grids for data collection. Such grid screening procedures are routinely used in the cryo-EM field.

a

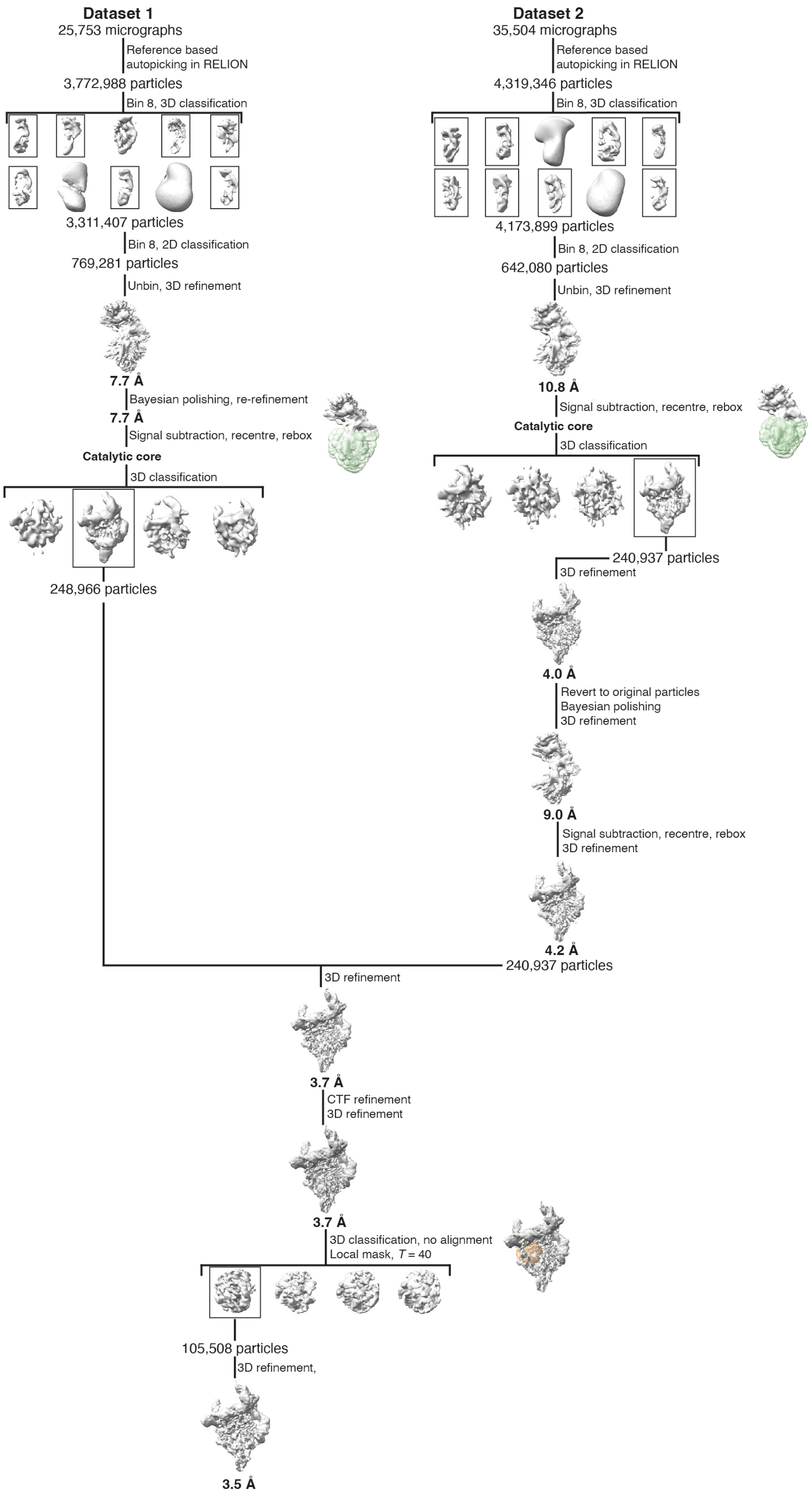

Dataset 1
25,753 micrographs
Reference based autopicking in RELION
3,772,988 particles
Bin 8, 3D classification
3,311,407 particles
Bin 8, 2D classification
769,281 particles
Unbin, 3D refinement
7.7 Å
Bayesian polishing, re-refinement
7.7 Å
Signal subtraction, recentre, rebox
Catalytic core
3D classification
248,966 particles
Dataset 2
35,504 micrographs
Reference based autopicking in RELION
4,319,346 particles
Bin 8, 3D classification
4,173,899 particles
Bin 8, 2D classification
642,080 particles
Unbin, 3D refinement
10.8 Å
Signal subtraction, recentre, rebox
Catalytic core
3D classification
240,937 particles
3D refinement
4.0 Å
Revert to original particles
Bayesian polishing
3D refinement
9.0 Å
Signal subtraction, recentre, rebox
3D refinement
4.2 Å
240,937 particles
3D refinement
3.7 Å
CTF refinement
3D refinement
3.7 Å
3D classification, no alignment
Local mask, T = 40
105,508 particles
3D refinement,
3.5 Å

b

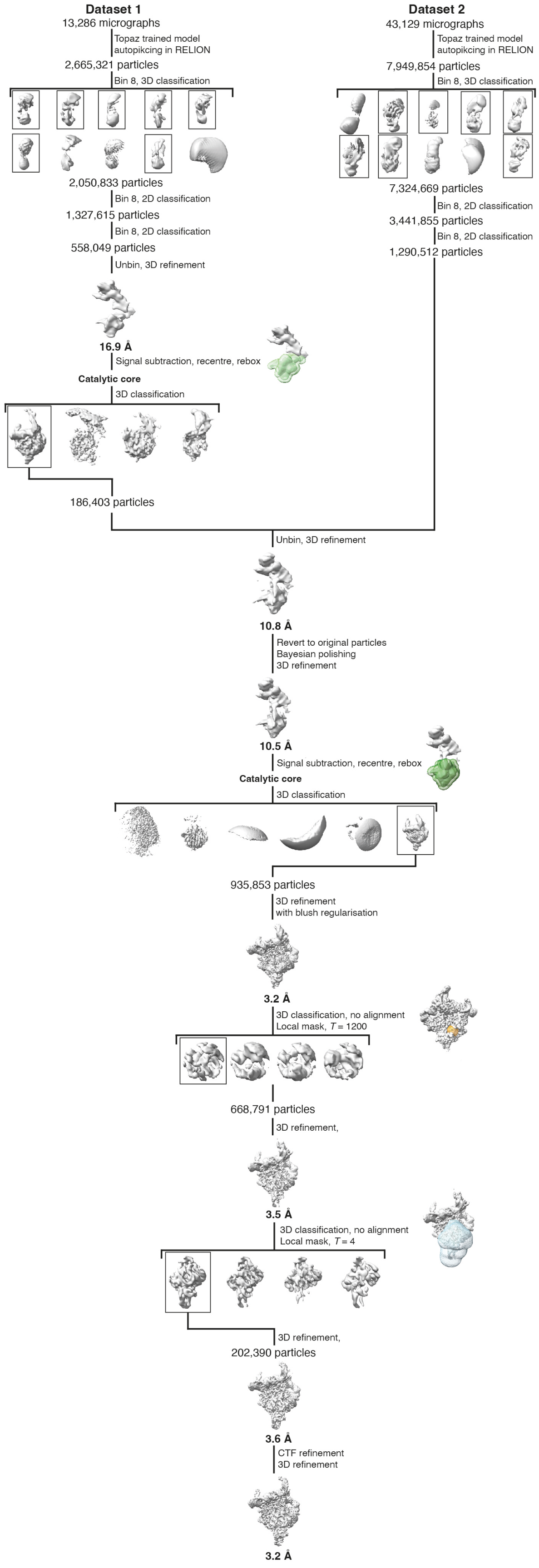
Dataset 1
13,286 micrographs
Topaz trained model
autopikcing in RELION
2,665,321 particles
Bin 8, 3D classification
2,050,833 particles
Bin 8, 2D classification
1,327,615 particles
Bin 8, 2D classification
558,049 particles
Unbin, 3D refinement
16.9 Å
Signal subtraction, recentre, rebox
Catalytic core
3D classification
186,403 particles
Dataset 2
43,129 micrographs
Topaz trained model
autopikcing in RELION
7,949,854 particles
Bin 8, 3D classification
7,324,669 particles
Bin 8, 2D classification
3,441,855 particles
Bin 8, 2D classification
1,290,512 particles
Unbin, 3D refinement
10.8 Å
Revert to original particles
Bayesian polishing
3D refinement
10.5 Å
Signal subtraction, recentre, rebox
Catalytic core
3D classification
935,853 particles
3D refinement
with blush regularisation
3.2 Å
3D classification, no alignment
Local mask, T = 1200
668,791 particles
3D refinement,
3.5 Å
3D classification, no alignment
Local mask, T = 4
3D refinement,
202,390 particles
3.6 Å
CTF refinement
3D refinement
3.2 Å

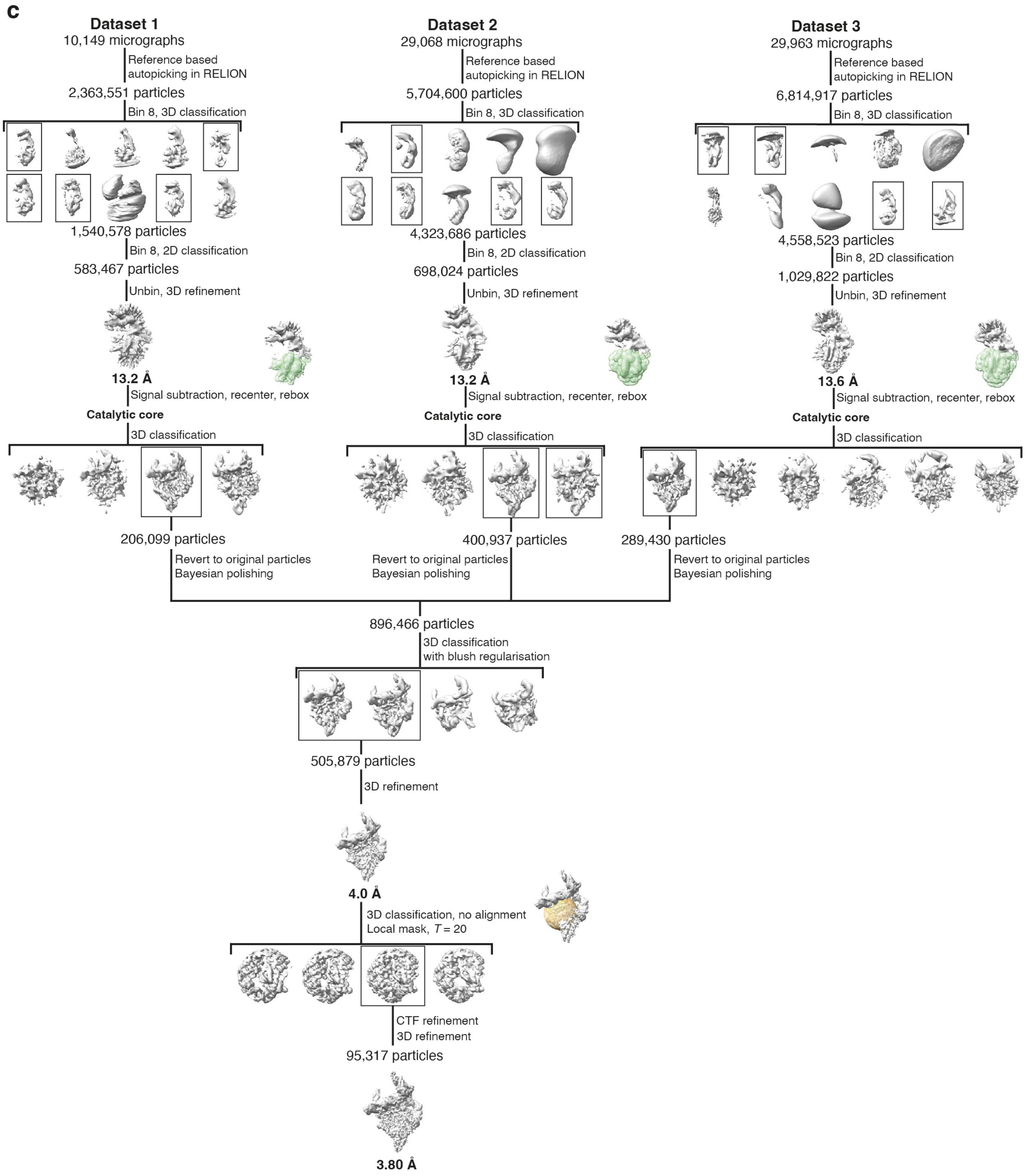

Supplementary Fig. 4 | Data processing strategies used to obtain reconstructions for the three telomerase states captured.

a–c, Data processing pipelines used for the initiation complex **(a)**, elongation complex **(b)**, and pre-termination complex **(c)**.

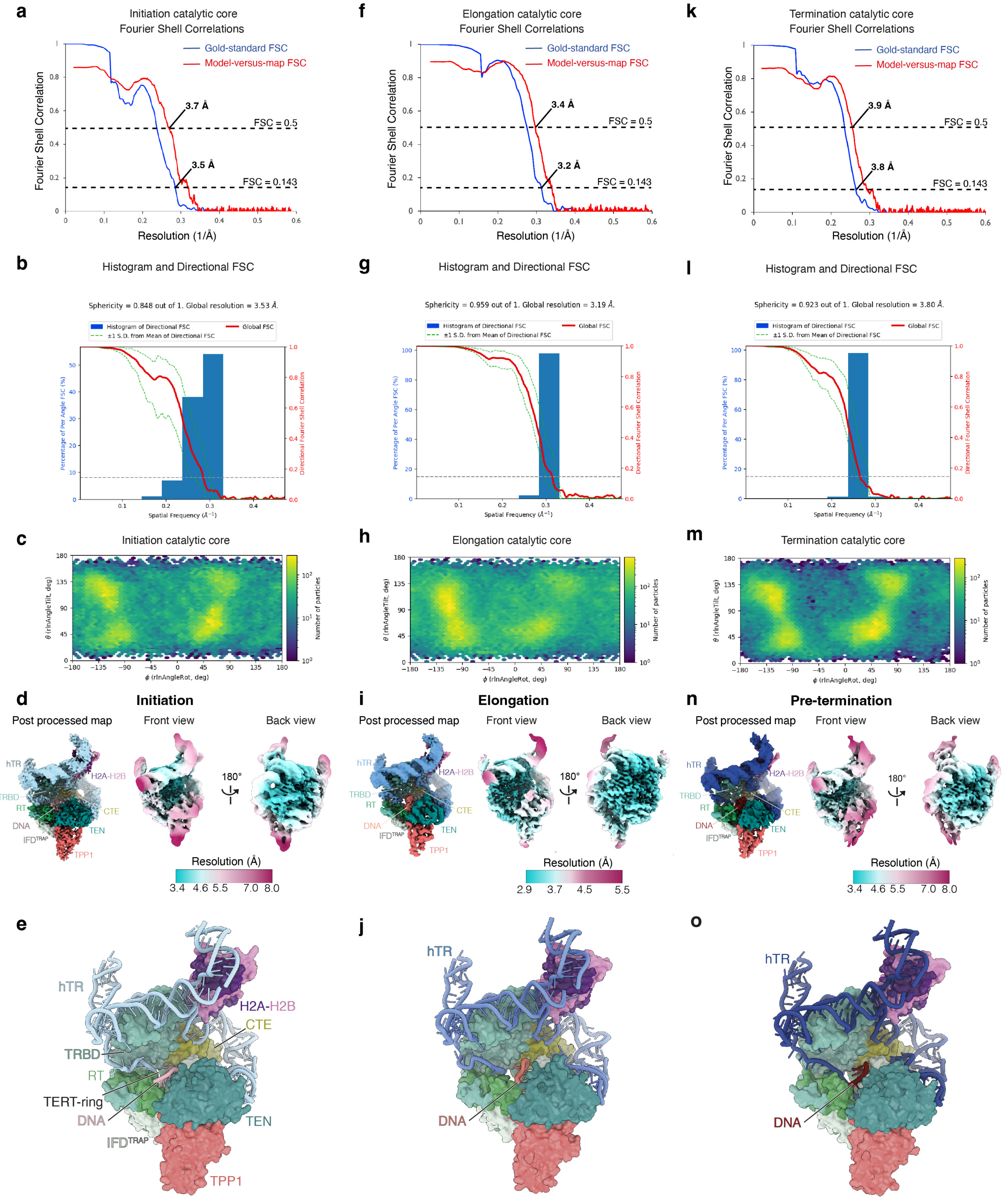

Supplementary Fig. 5 | Overall and local resolution estimates for the cryo-EM maps of the telomerase catalytic cores captured in the initiation, elongation, and pre-termination states.

a, f, k, Model-versus-map (red) and gold-standard (blue) FSC plots for telomerase initiation complex **(a)**, elongation complex **(f)**, and pre-termination complex **(k).** The resolution was estimated at FSC = 0.5 for model-versus-map FSCs and FSC = 0.143 for gold-standard FSC. **b, g, l,** Directional FSC plots and sphericity values are presented for the initiation **(b)**, elongation **(g)**, and pre-termination **(l)** complexes. Directional FSC plots were generated using a 3D-FSC server (https://3dfsc.salk.edu). **c, h, m,** 2D histograms depict the Euler angles of particles used for reconstructions of the telomerase initiation complex **(c)**, elongation complex **(h)**, and pre-termination complex **(m)**. Histograms were plotted using a Python script (https://githubhelp.com/Guillawme/angdist). **d, i, n,** Local resolution of the telomerase initiation complex **(d)**, elongation complex **(i)**, and pre-termination complex **(n)**. RELION 5.0 was used to generate local resolution estimation. Post-processed maps coloured and labelled according to domains are shown to demonstrate which regions of the complex are more flexible. **e, j, o** Structures of the telomerase catalytic core with the initiation DNA substrate **(e)**, elongation DNA substrate **(j)**, and pre-termination DNA substrate **(o)**. Proteins are shown in surface representation, nucleic acids are shown in cartoon style.

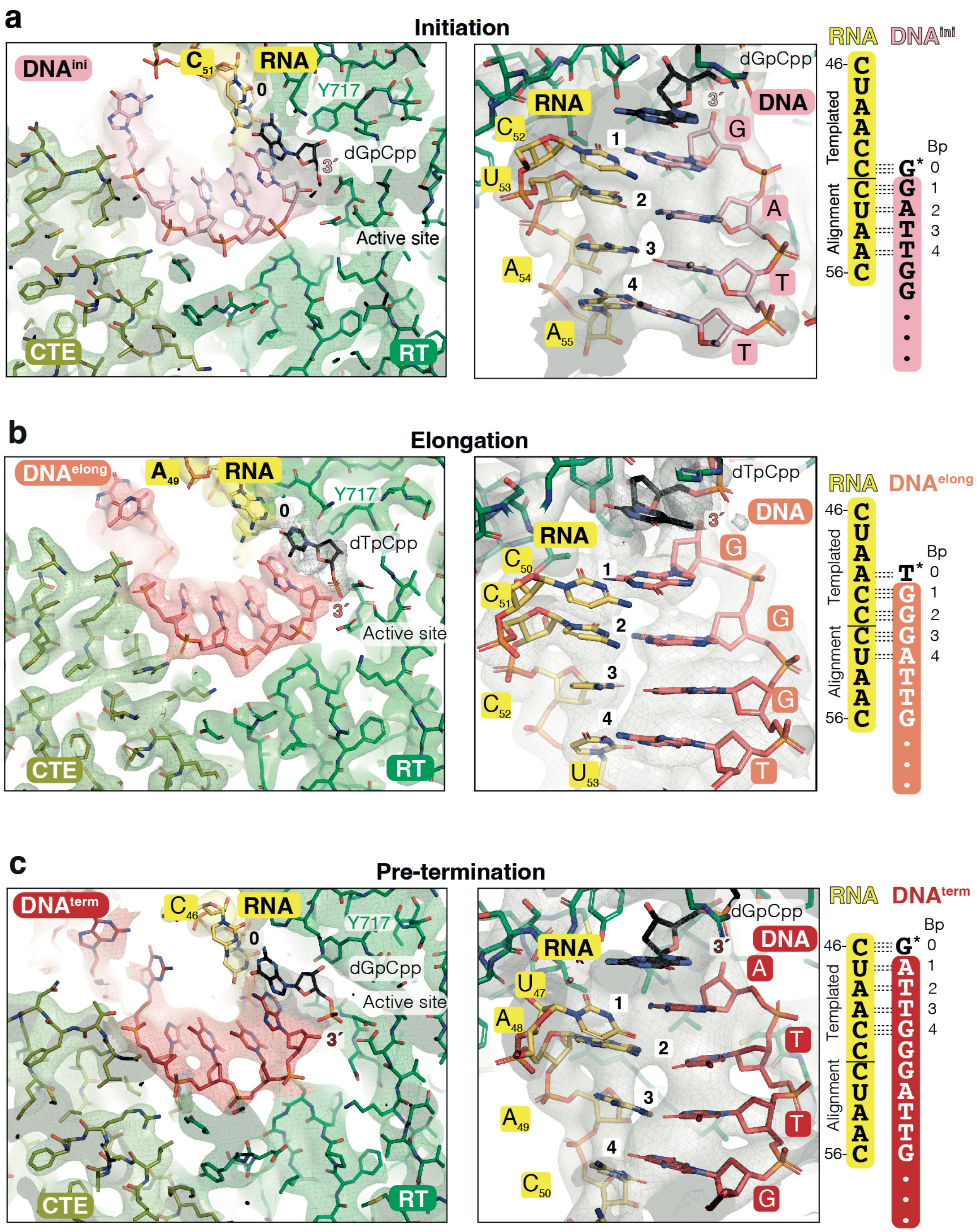

Supplementary Fig. 6 | Representative the map-model fit around the active site of telomerase for each complex.

a–c, Cryo-EM density of the telomerase active site and surrounding residues (left panel) and the RNA-DNA duplex (middle panel) with a schematic of the duplex formed for reference (right) in the initiation complex **(a)**, elongation complex **(b)**, and pre-termination complex **(c)**.

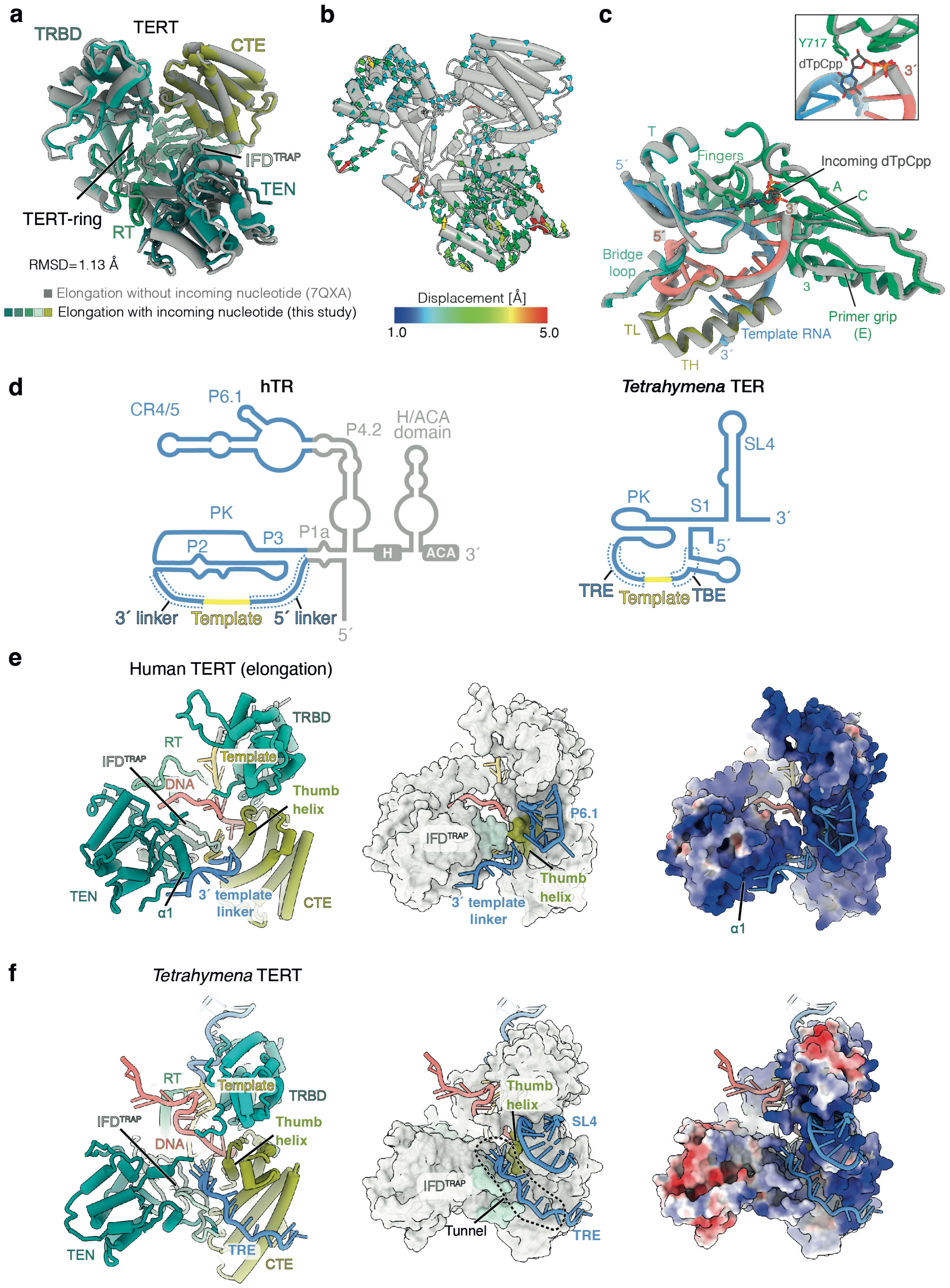
a
TRBD
TERT
CTE
IFDTRAP
TEN
TERT-ring
RT
RMSD=1.13 Å
Elongation without incoming nucleotide (7QXA)
Elongation with incoming nucleotide (this study)
b
Displacement [Å]
1.0
5.0
c
Y717
dTpCpp
3´
T
Fingers
Incoming dTpCpp
5´
A
C
3
5
Bridge loop
3
Primer grip (E)
TL
Template RNA
3´
TH
d
hTR
CR4/5
P6.1
P4.2
H/ACA domain
PK
P3
P1a
P2
H
ACA
3´
3´ linker
Template
5´ linker
5´
Tetrahymena TER
SL4
PK
S1
3´
5´
TRE
Template
TBE
e
Human TERT (elongation)
TRBD
RT
IFDTRAP
Template
DNA
Thumb helix
TEN
α1
3´ template linker
CTE
P6.1
IFDTRAP
Thumb helix
3´ template linker
α1
f
Tetrahymena TERT
RT
Template
TRBD
IFDTRAP
Thumb helix
DNA
TEN
TRE
CTE
Thumb helix
SL4
IFDTRAP
Tunnel
TRE

Supplementary Fig. 7 | Comparison of telomerase structures resolved in this study with previous structures of human telomerase and *Tetrahymena* telomerase.

a, Superposition of the telomerase elongation complex with (this study) and without (grey, PDB 7QXA[2]) an incoming dNTP. TERT domains are coloured according to Fig. 1d. **b,** Structural displacement of TERT from the binding of an incoming dNTP. The structure without incoming dNTP is shown in grey. Coloured vectors show displacement of this structure compared to our elongation complex with dNTP. **c,** Superposition of the conserved TERT motifs, hTR template and DNA substrates in the telomerase elongation complex with (this study) and without (grey, PDB 7QXA[2]) an incoming dNTP. Inset shows a close-up view of the region around the incoming nucleotide. TH, thumb helix; T, thumb loop. **d,** Comparison of the secondary structures of hTR (left) and *Tetrahymena* TER. **e,** Human telomerase with the RNA template and the 5′ and 3′ template linkers of hTR and DNA substrate. The 3′ template linker exits into an open space but passes close to the positively charged helix α1 in the TEN domain. Left, cartoon; centre, surface representation with TERT motifs close to hTR as it exits the active site; right, electrostatic surface potential of TERT showing the positively charged helix α1 (black line) close to the 3′ linker. In the central panel, P6.1 stem of hTR is also shown to demonstrate the proximity between the IFD^{TRAP}, the thumb helix and the 3′ template linker. **f,** *Tetrahymena* TERT with its template and the 5′ and 3′ flanking regions with DNA substrate (PDB 6D6V[4]). Left, cartoon representation; middle, surface representation of the TERT motifs proximal to the template recognition element (TRE) and a black dotted line showing the positively charged tunnel the TRE passes through; right, electrostatic surface potential of the TERT-ring showing the positively charged tunnel that the TRE passes through in *Tetrahymena*. This channel is closed on both sides, in contrast to the open space the 3′ linker of hTR exits into in human telomerase. In the central panel, stem loop 4 (SL4) of telomerase RNA, which is analogous to human P6.1 stem, is shown for comparison with the human structure (see **d** for their location in the RNA).

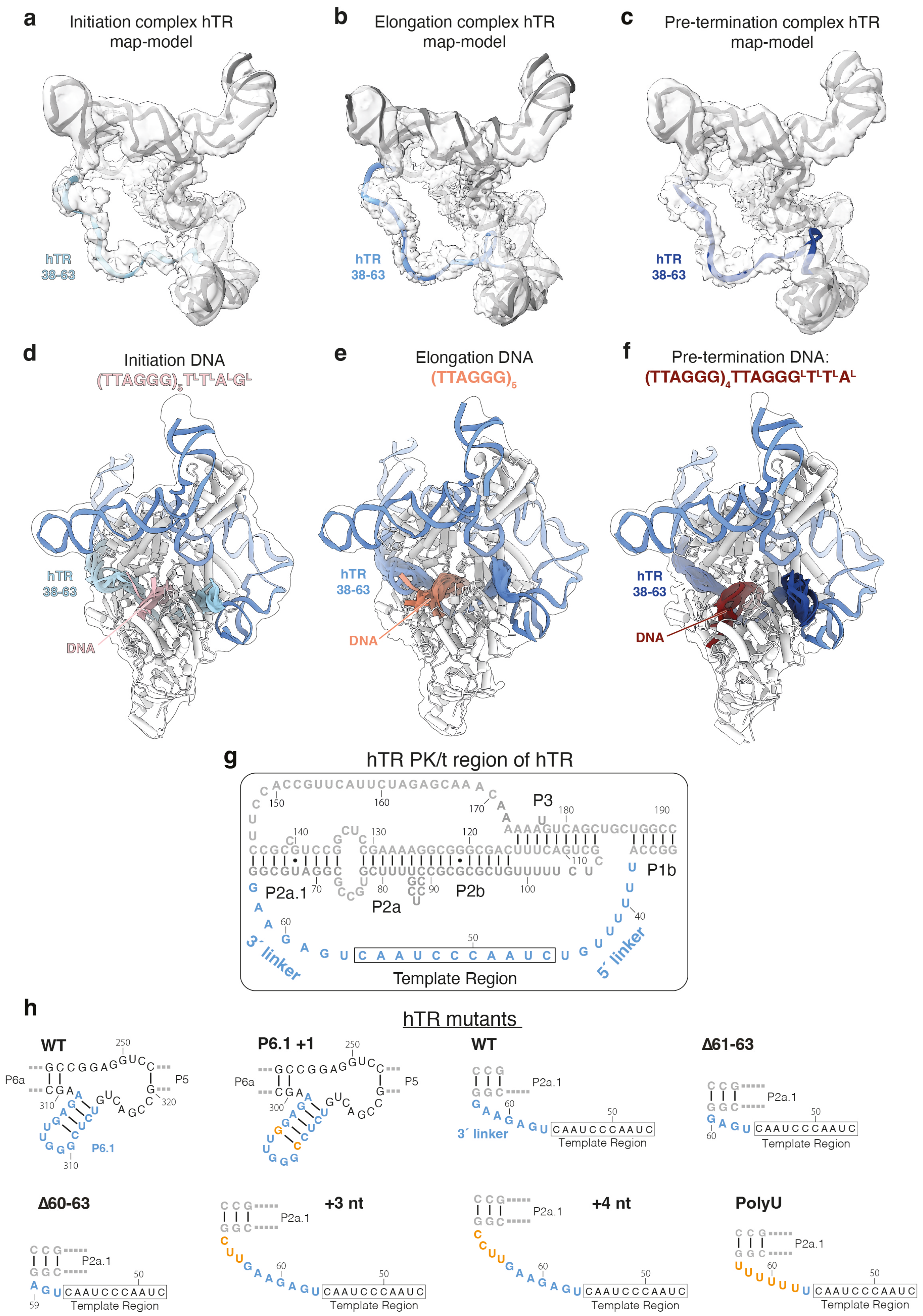
a
Initiation complex hTR map-model
hTR 38-63
b
Elongation complex hTR map-model
hTR 38-63
c
Pre-termination complex hTR map-model
hTR 38-63
d
Initiation DNA
(TTAGGG)5T^LT^LA^LG^L
hTR 38-63
DNA
e
Elongation DNA
(TTAGGG)5
hTR 38-63
DNA
f
Pre-termination DNA:
(TTAGGG)4TTAGGG^LT^LT^LA^L
hTR 38-63
DNA
g
hTR PK/t region of hTR
P3
P1b
P2a.1
P2a
P2b
3´ linker
5´ linker
CAAUCCCAAUC
Template Region
h
hTR mutants
WT
P6a
P5
P6.1
P6.1 +1
WT
P2a.1
3´ linker
Template Region
Δ61-63
Δ60-63
+3 nt
+4 nt
PolyU

Supplementary Fig. 8 | Characterisation of the 5′ and 3′ template linkers of hTR in the three catalytic states and schematics of hTR mutants.

a–c, Map-model fit of hTR in the initiation **(a)**, elongation **(b)** and pre-termination **(c)** complexes. The template and the 5′ and 3′ template linkers of hTR (nucleotides 38–63) are highlighted in different shades of blue, while the rest of hTR is coloured grey. **d–f,** Top 10 DRRAFTER models of the template and the 5′ and 3′ template linkers of hTR (nucleotides 38–63) in duplex with the DNA substrate are shown in the context of the full catalytic core model for the initiation complex **(d)**, elongation complex **(e)**, and pre-termination complex **(f)**. **g,** Schematic of the PK/t region of hTR present in the telomerase catalytic core. Regions of hTR modelled by DRRAFTER are coloured in blue. **h,** Schematics of the P6.1 and 3′ template linker hTR mutants tested in Fig. 3f. The schematic of the WT P6.1 and the 3′ template linker are also shown for comparison.

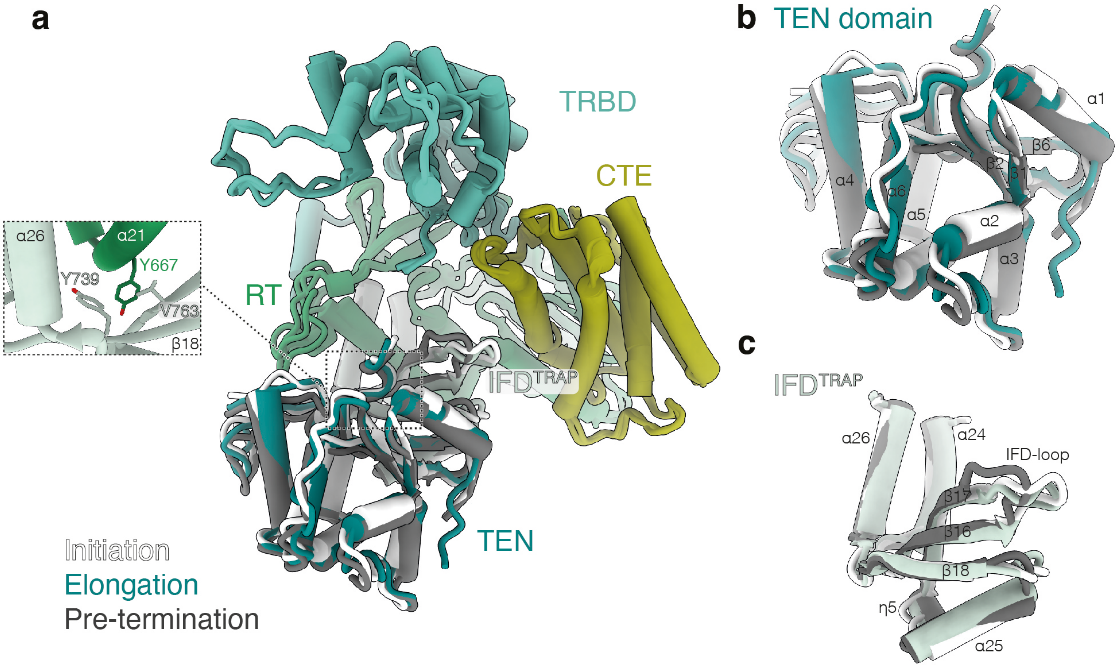

Supplementary Fig. 9 | Conformational flexibility of the TEN and IFD^{TRAP} domains of TERT. **a,** Cartoon representation of TERT in the three catalytic states captured in this study. The TRBD, RT and CTE domains of the three states are coloured as shown in Fig. 1d. The TEN domain and IFD^{TRAP} of the elongation complex is coloured as shown in Fig. 1d, whereas those of the initiation complex and pre-termination complex are coloured in white and grey, respectively. The superposition shows that the TEN domain and the IFD^{TRAP} exhibit greater structural differences across the 3 states compared to other domains of TERT. Inset shows a key stacking interaction between the base of the IFD^{TRAP} and the RT domain. This may restrict the movement of the IFD^{TRAP} relative to the TERT-ring through the catalytic cycle. **b,** Superposition of the TEN domains of the three telomerase complexes reported in this study. Colours are as described in **a**. **c,** Superposition of the IFD^{TRAP} domains of the 3 telomerase complexes reported in this study. Colours are as described in **a**.

Supplementary Table 1 | Cryo-EM data collection, refinement and validation statistics

	**Initiation catalytic core** (EMD-54920) (PDB 9SHY)	**Elongation catalytic core** (EMD-54921) (PDB 9SHZ)	**Pre-termination catalytic core** (EMD-54922) (PDB 9SI0)
**Data collection and processing**			
Magnification	Titan Krios G3i	Titan Krios G3i	Titan Krios G3i
Voltage (kV)	300	300	300
Electron exposure (e–/$Å^2$)	46–48	47–48	47–48
Defocus range (μm)	0.8–2.4	0.8–2.4	0.8–2.4
Pixel size (Å)	1.059	1.059	1.059
Symmetry imposed	C1	C1	C1
Initial particle images (no.)	8,092,334	10,615,175	10,422,787
Final particle images (no.)	105,508	202,390	95,317
Map resolution (Å)	3.5	3.2	3.8
FSC threshold	0.143	0.143	0.143
Map resolution range (Å)	3.4–8.0	2.9–5.5	3.4–8.0
**Refinement**			
Initial model used (PDB code)	7QXA	7QXA	7QXA
Model resolution (Å)	3.7	3.4	3.9
FSC threshold	0.5	0.5	0.5
Model resolution range (Å)	n/a	n/a	n/a
Map sharpening *B* factor ($Å^2$)	-50	-50	0
Model composition			
Non-hydrogen atoms	15,563	15611	15,566
Protein residues	1247	1250	1247
Nucleotides	263	264	263
Ligands	1: 1GC	1: A1A	1: 1GC
B factors ($Å^2$)			
Protein	215.75	143.10	198.92
Nucleotide	348.20	245.21	225.79
Ligand	195.81	166.33	229.63
R.m.s. deviations			
Bond lengths (Å)	0.006	0.006	0.007
Bond angles (°)	1.305	1.269	1.253
Validation			
MolProbity score	1.02 (100th)	1.07 (100th)	1.06 (100th)
Clashscore	2.41	2.85	2.75
Poor rotamers (%)	0	2	0
Ramachandran plot			
Favored (%)	99.27	99.02	98.70
Allowed (%)	0.65	0.98	1.30
Disallowed (%)	0.08	0.00	0.00

SUPPLEMENTARY REFERENCES

1. Ghanim, G.E. et al. Structure of human telomerase holoenzyme with bound telomeric DNA. *Nature* **593**, 449–453 (2021).
2. Sekne, Z., Ghanim, G.E., Roon, A.-M.M.v. & Nguyen, T.H.D. Structural basis of human telomerase recruitment by TPP1-POT1. *Science* **375**, 1173–1176 (2022).
3. Liu, B. et al. Structure of active human telomerase with telomere shelterin protein TPP1. *Nature* **604**, 578–583 (2022).
4. He, Y. et al. Structures of telomerase at several steps of telomere repeat synthesis. *Nature* **593**, 454–459 (2021).
