# Supplementary material for: Structures of nucleotide-bound human telomerase at several steps of its telomeric DNA repeat addition cycle
Source: Nat Commun. 2026 Jan 21;17:1847. doi: 10.1038/s41467-026-68560-8 (PMC12920920; doi:10.1038/s41467-026-68560-8)
Supplement: Supplementary file 2 — Description of Additional Supplementary Files [file 41467_2026_68560_MOESM2_ESM.pdf]

Description of Additional Supplementary Files

File name: Supplementary Data 1

Description: A list of mutations in TERT that have been characterised in vitro from either disease mutations or site-directed mutagenesis in biochemical studies, excluding the TEN domain and flexible linker between the TEN domain and TERT ring.

File name: Supplementary Data 2

Description: Pymol session containing DRRAFTER-modelled template linkers for the initiation state.

File name: Supplementary Data 3

Description: Pymol session containing DRRAFTER-modelled template linkers for the elongation state.

File name: Supplementary Data 4

Description: Pymol session containing DRRAFTER-modelled template linkers for the pre-termination state.

File name: Supplementary Data 5

Description: Sequences of DNA primers used for mutagenesis of TERT and hTR.
