# Supplementary material for: Structures of nucleotide-bound human telomerase at several steps of its telomeric DNA repeat addition cycle
Source: Nat Commun. 2026 Jan 21;17:1847. doi: 10.1038/s41467-026-68560-8 (PMC12920920; doi:10.1038/s41467-026-68560-8)
Supplement: Supplementary file 9 — Transparent Peer Review file [file 41467_2026_68560_MOESM9_ESM.pdf]

nature portfolio

Peer Review File

Structures of nucleotide-bound human telomerase at several steps of its telomeric DNA repeat addition cycle

Corresponding Author: Dr Thi Hoang Duong (Kelly) Nguyen

This file contains all reviewer reports in order by version, followed by all author rebuttals in order by version.

Version 0:

Reviewer comments:

Reviewer #1

(Remarks to the Author)
Balch et al. report multiple cryo-EM structures of the human telomerase during the nucleotide addition cycle. As expected from the Nguyen lab, the here presented biochemical and structural data is of high quality. The findings are important for our general understanding of telomerase biology and contribute an important next step in further elucidating the fundamental mechanism of nucleotide addition. The manuscript is well written and the figures are clear. I recommend publication of this manuscript in Nature Communications.

The authors should, however, extend their methods section. Description of the telomerase reconstitution and purification is extremely short. It becomes extremely tedious to follow all cited references in this section and the way this paragraph of the method section is written obfuscates reproducibility. Therefore, the authors must write an extended methods section to adequately describe the state-of-the-art experimental procedures for the reconstitution and purification of the telomerase including precise DNA sequences used. Additionally, the authors should also make the code available used for plotting the conformational changes.

Reviewer #2

(Remarks to the Author)
This manuscript reports three new cryo-EM structures of human telomerase (3.2, 3.5, and 3.8 Å), catching the enzyme/primer/incoming nucleotide complex at three stages of catalysis: initiation, elongation, and pre-termination. The structural analysis is of high quality. One important new finding is that the TRAP-wedge and the TEN N-terminal helix bind the DNA and RNA. However, in light of previous structural analysis of human telomerase from this group and others, decades-long biochemical and biophysical analyses of human and Tetrahymena telomerase, and the resolution (medium resolution at best) of the presented structures, the increase in knowledge of the telomerase reaction mechanism from the remaining experiments is not significant.
Major comments
1. It is not clear what new knowledge is gained from the cartoons and 3D structures in Figures 1 and 2. These figures contain information available from previous cryo-EM structures at similar or even better resolution from this lab, Juli Feigon's lab, and Ming Lei's lab.
2. Figure 3 describes how a 4-5 bp template-primer complex forms regardless of the sequence of the duplex region, which has been observed in previous structures of human telomerase and Tetrahymena telomerase, and suggested by previous telomerase activity and single molecule studies of both human and Tetrahymena telomerase (example: Julian J-L Chen lab's studies).
3. The reason for how a constant RNA-DNA hybrid is maintained has been demonstrated previously from the human cryo-EM structures at similar resolution from the Lei lab. In a study published in Cell Research (Wan et al, 2021, Zipper head mechanism of telomere synthesis by human telomerase), the authors describe how TERT L980 "zipper head" acts to unzip old RNA-DNA base pairing when new bases are added to the 3' end of DNA. There is a figure panel in that study that is similar to Figure 3 (c, e and g) panels in this manuscript and Wan et al also propose this as part of their final model in the paper. So, in addition to visualizing the incoming nucleotide, Figure 3 of the manuscript under review doesn't add much new insight.

4. Figure 4 gives a name for the regions flanking the RNA template as 5’ and 3’ linkers, but the importance of these regions and the model for elongation and contraction of these regions as nucleotide addition proceeds is well appreciated already, as is noted by the authors. It might be argued that this study provides a structural basis for the model. But is this a strong argument, given the low to medium resolution of the structures and the heavy processing (DRRAFTER modeling) needed to bring out these linker regions? The map-model fit for the linkers - as shown for the RNA-DNA in the fifth supplementary figure - should be shown for the reader to assess the quality of the modeled linkers.
5. In a recent publication from this lab, dimerization of telomerase is suggested to be important for assembly of the enzyme but the authors did not rule out contributions to activity. Did the authors pick dimeric particles in their nucleotide bound structures to ask if the dimer is compatible with nucleotide binding? What changes occur in the dimer upon nucleotide binding that would favor, disfavor, or not affect the active site configuration? These seem like important follow-up questions to address for that publication that the current study is well suited to address.
6. TPT increases telomerase processivity. Comparative analysis of dNTP-bound telomerase and telomerase-TPT should be performed to differentiate between features coming from telomerase and those from bound TPT.

Other suggestions:

1. Consider moving figures 1-3 to the supplement based on comments above.
2. The manuscript uses new terminology for 5’ and 3’ linkers in the RNA. These need to be defined in the context of the template boundary element (TBE) and template recognition element (TRE). TBE and TRE should be shown in Figure 4A for example.
3. The model in figure 6 is quite similar to the analogous model figure in the Cell Research cryo-EM paper of the Lei lab, with the exception of the TRAP wedge. Consider moving this figure to the supplement unless the point of the figure is the TRAP wedge (in which case the figure should make that clear).

Reviewer #3

(Remarks to the Author)
The study presented by Balch et al represents a structure-focused study that contributes to the understanding of telomerase function, most notably the steps involved in repeat addition processivity. The authors produce three high quality structures of the catalytic core of the telomerase holoenzyme in complex with substrate DNA and incoming nucleotide at three different stages of telomere extension: initiation, elongation, and termination. These structures demonstrate that the telomerase RNA template binds to substrate DNA through a constant 4-5 base RNA-DNA duplex and confirms that L980 functions as a "zipper head," maintaining the 4-5 base duplex as the substrate is extended. Additionally, the authors demonstrated mechanisms through which the 3' and 5' flanking regions of the RNA template affect telomerase activity and the role of the TEN domain in DNA substrate binding. In general, the study is well conceived, organized, executed, and communicated. The manuscript does a particularly good job of integrating the new findings presented with published work to, collectively, provide needed explanations and interpretations related to the nuances of telomerase structure and function. In our opinion, the manuscript is acceptable for publication. However, we include some suggestions and comments for the authors to consider when revising their manuscript.

Minor Critiques:

Intro:
It would be helpful to introduce some of the unique features of TERT that contribute to RAP in the intro, based on what is already known. While it does come up later in the results, the third paragraph of the intro would be a good place to expand on the roles of RBD, TEN, and the CTE. This inclusion would also help to support the following paragraph that discusses the limitations of using T. castaneum TERT that lacks several of these unique features, as is already stated.

Line 95: “locked nucleic acid (LDNA) residues…” Residues is probably not the best term here. Perhaps “analogs” or “modifications” is better.

Methods:
Regarding the telomerase extension assays: Is there no salt (NaCl or KCl) in the telomerase activity assays? Secondly, is the use of a one-tailed t-test for statistical analysis the most appropriate for these studies? It is suggested that these statistics be redone using a two-tailed t-test.

Could the authors provide any information regarding the unexplained bands at 40kDa, and 55kDa, as well as several higher molecular weight bands around 130kDa in the initiation complex that are not present (or as apparent) in other purifications (Extended Fig 1b)?

Please include more details regarding the specific ratios/amounts of pcDNA3.1-ZZ-TEV-twin-Strep-SUMO*-TERT and pcDNA3.1-U3-hTR-HDV as well as the additives used including PEI, glucose, sodium butyrate, VPA, etc. for transfection enhancement and optimized expression.

Reviewer #4

(Remarks to the Author)

I co-reviewed this manuscript with one of the reviewers who provided the listed reports. This is part of the Nature Communications initiative to facilitate training in peer review and to provide appropriate recognition for Early Career Researchers who co-review manuscripts.

Version 1:

Reviewer comments:

Reviewer #1

(Remarks to the Author)
The authors have sufficiently addressed my concerns.

Reviewer #2

(Remarks to the Author)
The authors have responded to the earlier critiques with clarifications, arguments, and reorganization of figures, and some additional experiments. Although there is subjectivity in judging the impact of a study, I still do not interpret the findings as carrying the level of significance/novelty (justifying publication in Nature Comm.) that the authors emphasize. That said, I do not offer any criticism of the quality or quantity of the data. For that reason, I do not request additional revisions or further experiments.

Reviewer #3

(Remarks to the Author)
The authors have addressed all of my concerns in the revised manuscript.

Reviewer #4

(Remarks to the Author)
I co-reviewed this manuscript with one of the reviewers who provided the listed reports. This is part of the Nature Communications initiative to facilitate training in peer review and to provide appropriate recognition for Early Career Researchers who co-review manuscripts.

Open Access This Peer Review File is licensed under a Creative Commons Attribution 4.0 International License, which permits use, sharing, adaptation, distribution and reproduction in any medium or format, as long as you give appropriate credit to the original author(s) and the source, provide a link to the Creative Commons license, and indicate if changes were made.
In cases where reviewers are anonymous, credit should be given to 'Anonymous Referee' and the source.
The images or other third party material in this Peer Review File are included in the article's Creative Commons license, unless indicated otherwise in a credit line to the material. If material is not included in the article's Creative Commons license and your intended use is not permitted by statutory regulation or exceeds the permitted use, you will need to obtain

permission directly from the copyright holder.
To view a copy of this license, visit https://creativecommons.org/licenses/by/4.0/

We thank all reviewers for their careful reading and thoughtful comments of our manuscript. We have carefully studied every point raised, performed additional experiments to dissect the contribution of TPP1-POT1-TIN2 (TPT) and incoming nucleotide on telomerase activity, and revised the manuscript based on the comments. We highlight all the changes in the manuscript. Below is our point-by-point response to the reviewers' comments:

Reviewer #1 (Remarks to the Author):
Balch et al. report multiple cryo-EM structures of the human telomerase during the nucleotide addition cycle. As expected from the Nguyen lab, the here presented biochemical and structural data is of high quality. The findings are important for our general understanding of telomerase biology and contribute an important next step in further elucidating the fundamental mechanism of nucleotide addition. The manuscript is well written and the figures are clear. I recommend publication of this manuscript in Nature Communications.

We appreciate the positive remarks from the reviewer.

The authors should, however, extend their methods section. Description of the telomerase reconstitution and purification is extremely short. It becomes extremely tedious to follow all cited references in this section and the way this paragraph of the method section is written obfuscates reproducibility. Therefore, the authors must write an extended methods section to adequately describe the state-of-the-art experimental procedures for the reconstitution and purification of the telomerase including precise DNA sequences used. Additionally, the authors should also make the code available used for plotting the conformational changes.

We thank the reviewer for this helpful suggestion. We have now expanded the Materials & Methods section to include a detailed description of the telomerase reconstitution and purification procedures, including the precise DNA sequences used.

Regarding the code used for plotting the conformational changes, as mentioned in the Acknowledgment, the code was shared with us by Prof. Jamie Cate (University of California, Berkeley). It would not be appropriate for us to share the code with the paper as we do not own the code. However, Prof. Cate is willing for us to share the code through email contact. We have added a phrase in the Author Information section to notify the reader of this information.

Reviewer #2 (Remarks to the Author):

This manuscript reports three new cryo-EM structures of human telomerase (3.2, 3.5, and 3.8 Å), catching the enzyme/primer/incoming nucleotide complex at three stages of catalysis: initiation, elongation, and pre-termination. The structural analysis is of high quality. One important new finding is that the TRAP-wedge and the TEN N-terminal helix bind the DNA and RNA. However, in light of previous structural analysis of human telomerase from this group and others, decades-long biochemical and biophysical analyses of human and Tetrahymena telomerase, and the resolution (medium resolution at best) of the presented structures, the increase in knowledge of the telomerase reaction mechanism from the remaining experiments is not significant.

Major comments
1. It is not clear what new knowledge is gained from the cartoons and 3D structures in Figures 1 and 2. These figures contain information available from previous cryo-EM structures at similar or even better resolution from this lab, Juli Feigon's lab, and Ming Lei's lab.

We thank the reviewer for the suggestions. We appreciate that structures of telomerase have been reported previously from our lab (Ghanim et al., 2021), Ming Lei's lab (Wan et al., 2021), and Juli Feigon's lab (Liu et al., 2022). However, all of these structures were resolved with the same DNA substrate permutation ending in -TTAGGG, and no incoming dNTP. Our study presents the first structure with the -TTAGGG substrate permutation that also includes an incoming nucleotide. Moreover, this is the first report of the human telomerase catalytic core captured in the initiation and pre-termination states using substrate permutations ending in -TTAG, and -TTA, respectively. To improve clarity and avoid redundancy, we have now combined Figures 1 and 2 into a single figure (now Fig. 1) and moved some of the additional panels to Extended Data Figs. 1 and 2.

2. Figure 3 describes how a 4-5 bp template-primer complex forms regardless of the sequence of the duplex region, which has been observed in previous structures of human telomerase and Tetrahymena telomerase, and suggested by previous telomerase activity and single molecule studies of both human and Tetrahymena telomerase (example: Julian J-L Chen lab's studies).

We understand the point raised by the reviewer. The thorough research on *Tetrahymena* telomerase from Juli Feigon's lab (He et al., 2021), which we cite in our manuscript, allows for an interesting comparison with our findings for human telomerase in this study. As seen in this study, it was important to experimentally obtain structures of the Tetrahymena enzyme with different DNA substrates in order to understand its mechanism of repeat synthesis.

The reviewer correctly points out that the duplex length in both human and *Tetrahymena* telomerase 4-5 base pairs in length. However, humans and ciliates are evolutionarily distant. Both TERT and TR have diverged substantially. Therefore, the findings for *Tetrahymena* telomerase (He et al., 2021) may not be directly extrapolated to human telomerase. There are some significant differences which we state in the manuscript. For example, we show in Extended Data Fig. 7e and f how the path of the TRE in *Tetrahymena* differs significantly from the path of the 3′ linker in human telomerase. Therefore, our study is essential to the understanding of human telomerase's mechanism of action.

As the reviewer mentions, extensive biochemical work aiming at understand the catalytic cycle of human telomerase has been published. Until now, it was still an outstanding question whether the DNA-RNA duplex length in the telomerase remained constant or if it increased as repeat synthesis occurred. Research from Wu et al., (2014) suggested that duplex length increased with telomeric repeat synthesis. Bley et al., (2012) showed human telomerase can extend a 5 base pair RNA-DNA duplex added in *trans* more efficiently than longer duplexes. However, to our knowledge, none of these had directly demonstrated that a 4bp duplex is formed between the telomeric DNA and template RNA.

Through capturing structural snapshots of human telomerase at distinct steps of the catalytic cycle, each also with the incoming nucleotide, our work provides direct evidence for the duplex length through the catalytic cycle. Our study, therefore, answers the uncertainty regarding the human telomerase duplex length throughout its catalytic cycle. At the same time, it also puts much of the previous biochemical work mentioned above into context as discussed in our paper and remarked by Reviewer 3.

3. The reason for how a constant RNA-DNA hybrid is maintained has been demonstrated previously from the human cryo-EM structures at similar resolution from the Lei lab. In a study published in Cell Research (Wan et al, 2021, Zipper head mechanism of telomere synthesis by human telomerase), the authors describe how TERT L980 "zipper head" acts to unzip old RNA-DNA base pairing when new bases are added to the 3' end of DNA. There is a figure panel in that study that is similar to Figure 3 (c, e and g) panels in this manuscript and Wan et

al also propose this as part of their final model in the paper. So, in addition to visualizing the incoming nucleotide, Figure 3 of the manuscript under review doesn't add much new insight.

We thank the reviewer for this comment. We acknowledge the important contribution of Wan et al., (2021) study. However, it should be noted that they suggested that Leu980 limits the RNA-DNA duplex length based on computational modelling only. They simulate how Leu980 would interact with different base pairs at different stages of the catalytic cycle. Only the structure of human telomerase with a DNA substrate ending in the -TTAGGG permutation was experimentally determined in this work.

In our manuscript, we experimentally determined three structures of the human telomerase catalytic core with different substrate permutations. In agreement with the computational modelling performed by Wan et al., (2021), our structures confirm that human telomerase limits the RNA-DNA duplex length in its active site. Therefore, we believe Fig. 3 (Fig. 2 in the revised manuscript) is a strong validation of the molecular dynamics stimulation by Wan et al (2021). Furthermore, we were able to observe the differences in the duplex stability among the three DNA substrates, which could not be elucidated through molecular dynamics. This provides an explanation for the differential binding affinity of the different DNA permutations to telomerase.

The accompanying schematics in Fig. 2 in the revised manuscript further illustrate the conservation of RNA-DNA duplex and depict how the overall architecture of the active site remains largely constant across the three different catalytic states captured. We consider this a key part of the manuscript that helps guide the reader through the different catalytic states and facilitates the overall understanding of the paper.

4. Figure 4 gives a name for the regions flanking the RNA template as 5' and 3' linkers, but the importance of these regions and the model for elongation and contraction of these regions as nucleotide addition proceeds is well appreciated already, as is noted by the authors. It might be argued that this study provides a structural basis for the model. But is this a strong argument, given the low to medium resolution of the structures and the heavy processing (DRRAFTER modeling) needed to bring out these linker regions? The map-model fit for the linkers - as shown for the RNA-DNA in the fifth supplementary figure - should be shown for the reader to assess the quality of the modeled linkers.

We appreciate the reviewer's concern regarding the resolution of the map at the linker regions. As stated in the manuscript, the flexible nature of the ssRNA 5′ and 3′ linkers limits the resolution of these areas, as indicated by the local resolution estimates (Extended Data Fig. 5d, i, n).

In both cryo-EM and X-ray crystallography, it is standard practice to present and deposit one map and one corresponding model for each structure. Conventional cryo-EM workflows generate a single map filtered to a consensus resolution determined by the gold-standard Fourier Shell Correlation (FSC) criterion. Consequently, density for flexible regions typically appears weaker in this consensus map. When we generated a map filtered to local resolution, the densities for the linker regions appeared clear and continuous, though with lower-resolution features. To model these regions, we lowered the map threshold and applied map blurring in Coot, ensuring that the backbone density remained consistent with the map.

The resulting models were refined using standard model refinement protocols. During refinement, B-factors (temperature factors) are assigned to each residue, with higher B-factors reflecting greater flexibility, thus providing another representation of molecular dynamics within the model.

While these procedures are routine, they inherently yield only a single structural representation of the ssRNA linkers. This static depiction cannot fully capture the conformational variability of

these regions. To better address this limitation, we collaborated with the laboratory of Rhiju Das, experts in RNA modeling. They generated an ensemble of the ten most probable conformations for the ssRNA linkers and the RNA template interacting with the DNA substrate (Extended Data Fig. 8d–f). We believe this ensemble approach is valuable for depicting the intrinsic flexibility of the ssRNA linkers beyond the conventional one-map–one-model paradigm.

Following the reviewer's suggestion, we have now included map–model fits for the consensus model of the ssRNA region of hTR (nucleotides 38–63), encompassing the 5′ linker, template region, and 3′ linker, in Extended Data Fig. 8a–c.

5. In a recent publication from this lab, dimerization of telomerase is suggested to be important for assembly of the enzyme but the authors did not rule out contributions to activity. Did the authors pick dimeric particles in their nucleotide bound structures to ask if the dimer is compatible with nucleotide binding? What changes occur in the dimer upon nucleotide binding that would favor, disfavor, or not affect the active site configuration? These seem like important follow-up questions to address for that publication that the current study is well suited to address.

We thank the reviewer for this thoughtful comment. The data that yielded the dimer structure came from these datasets we present in this study. However, the telomerase dimer is incredibly rare and required substantial data processing to isolate (Balch et al., 2025). We also had to perform extensive classification to obtain the nucleotide-bound state for each structure presented in this manuscript (Extended Data Fig. 4a-c). Therefore, we did not have enough particles representing the dimer or the catalytic core in a nucleotide-bound state to perform a detailed structural analysis. Nonetheless, based on the available structures, the catalytic cores of dimeric human telomerase appear to be compatible with nucleotide binding, although we did not directly observe this.

6. TPT increases telomerase processivity. Comparative analysis of dNTP-bound telomerase and telomerase-TPT should be performed to differentiate between features coming from telomerase and those from bound TPT.

We thank the reviewer for this nice suggestion. We have now included telomerase activity assays comparing the effects of the dNTPs used in each reconstitution with/without TPT in Extended Data Fig. 1c-f. Our results show that the stimulation of telomerase activity by the dNTP is mild, whereas the stimulation by TPT is much greater. Therefore, when adding both dNTP and TPT to telomerase, the enhancement in processivity is dominated by the effects of TPT (Extended Data Fig. 1c-f).

Other suggestions:

1. Consider moving figures 1-3 to the supplement based on comments above.

As mentioned above, we have combined fig. 1 and 2 into one single figure, now Fig. 1. Regarding Fig. 3, we believe it guides the reader through the catalytic states and supports a better clarity of the paper, as explained in the response to point 3 made by the reviewer.

2. The manuscript uses new terminology for 5' and 3' linkers in the RNA. These need to be defined in the context of the template boundary element (TBE) and template recognition element (TRE). TBE and TRE should be shown in Figure 4A for example.

We thank the reviewer for this thoughtful comment. While the TBE and TRE play crucial roles in defining the template boundary in *Tetrahymena* telomerase (Miller & Collins, 2002). We believe referring to the 5′ linker as the TBE could be confusing because in human telomerase,

the P1b helix located upstream of the 5′ linker also contributes to defining the template boundary (Chen & Greider, 2003).

Although we have drawn parallels between human and *Tetrahymena* telomerase where appropriate, the 5′ and 3′ template-flanking regions in hTR have diverged substantially in both sequence and secondary structure from the TBE and TRE of *Tetrahymena* telomerase (Wu et al., 2017). We further highlight the distinct paths of the TRE in *Tetrahymena* and the 3′ linker in human telomerase in Extended Data Fig. 7e,f. To help with the comparison between hTR and *Tetrahymena* TER, we have made a new figure panel in Extended Data Fig. 7d to show the TRE and TBE in Tetrahymena and the analogous regions in hTR. We also add the phrase “analogous to the Tetrahymena TBE/TRE” when we discussed the 5′ and 3 template linkers in the revised manuscript.

3. The model in figure 6 is quite similar to the analogous model figure in the Cell Research cryo-EM paper of the Lei lab, with the exception of the TRAP wedge. Consider moving this figure to the supplement unless the point of the figure is the TRAP wedge (in which case the figure should make that clear).

We are grateful for the reviewer’s suggestion. We have revised the figure legend to clearly emphasize that the coordinated roles of the TRAP wedge, α1 helix, and DNA anchor site of TERT are the primary focus of this figure.

Reviewer #3 (Remarks to the Author):

The study presented by Balch et al represents a structure-focused study that contributes to the understanding of telomerase function, most notably the steps involved in repeat addition processivity. The authors produce three high quality structures of the catalytic core of the telomerase holoenzyme in complex with substrate DNA and incoming nucleotide at three different stages of telomere extension: initiation, elongation, and termination. These structures demonstrate that the telomerase RNA template binds to substrate DNA through a constant 4-5 base RNA-DNA duplex and confirms that L980 functions as a "zipper head," maintaining the 4-5 base duplex as the substrate is extended. Additionally, the authors demonstrated mechanisms through which the 3' and 5' flanking regions of the RNA template affect telomerase activity and the role of the TEN domain in DNA substrate binding. In general, the study is well conceived, organized, executed, and communicated. The manuscript does a particularly good job of integrating the new findings presented with published work to, collectively, provide needed explanations and interpretations related to the nuances of telomerase structure and function. In our opinion, the manuscript is acceptable for publication. However, we include some suggestions and comments for the authors to consider when revising their manuscript.

We are grateful for the positive remarks and the appreciation of how this work integrates with a wealth of previous research in the field.

Minor Critiques:

Intro:
It would be helpful to introduce some of the unique features of TERT that contribute to RAP in the intro, based on what is already known. While it does come up later in the results, the third paragraph of the intro would be a good place to expand on the roles of RBD, TEN, and the CTE. This inclusion would also help to support the following paragraph that discusses the limitations of using T. castaneum TERT that lacks several of these unique features, as is already stated.

We thank the reviewer for this helpful suggestion. We have now modified the introduction to include the unique features of TERT that contribute to RAP. Specifically, we have expanded the third paragraph to include the roles of the RBD, TEN and the CTE domains.

Line 95: “locked nucleic acid (LDNA) residues…” Residues is probably not the best term here. Perhaps “analogs” or “modifications” is better.

We agree and have now replaced “locked nucleic acid (LDNA) residues” with “locked nucleic acid analogs”.

Methods:
Regarding the telomerase extension assays: Is there no salt (NaCl or KCl) in the telomerase activity assays?

We thank the reviewer for pointing this out. We have now made sure all reaction buffer components, including the type of salt used and its concentration, are included in the Materials &Methods section.

Secondly, is the use of a one-tailed t-test for statistical analysis the most appropriate for these studies? It is suggested that these statistics be redone using a two-tailed t-test.

We appreciate this suggestion. We have redone all statistical analyses using a two-tailed t-test. In the revised figures, *P*-values are indicated using asterisks rather than numerical values.

Could the authors provide any information regarding the unexplained bands at 40kDa, and 55kDa, as well as several higher molecular weight bands around 130kDa in the initiation complex that are not present (or as apparent) in other purifications (Extended Fig 1b)?

We understand the reviewer’s concern. The unexplained bands observed in the initiation complex but not in the elongation and pre-termination complex purifications likely result from a variability in the silver staining procedure. Depending on the staining intensity, some non-specific bands can appear more prominent in some reconstitutions than others. Upon close observation, there are also faint bands at 40, 55, and 130 kDa the silver stained SDS-PAGE gels of the elongation and pre-termination complexes. With further staining of these gels, the bands would become more prominent, like in the initiation complex SDS-PAGE gel.

Please include more details regarding the specific ratios/amounts of pcDNA3.1-ZZ-TEV-twin-Strep-SUMO*-TERT and pcDNA3.1-U3-hTR-HDV as well as the additives used including PEI, glucose, sodium butyrate, VPA, etc. for transfection enhancement and optimized expression.

We thank the reviewer for this suggestion. We have now added detailed information regarding the specific ratios and amounts of pcDNA3.1-ZZ-TEV-twin-Strep-SUMO*-TERT and pcDNA3.1-U3-hTR-HDV, as well as additives used for the transfection in the extended Methods section.

Reviewer #4 (Remarks to the Author):

I co-reviewed this manuscript with one of the reviewers who provided the listed reports. This is part of the Nature Communications initiative to facilitate training in peer review and to provide appropriate recognition for Early Career Researchers who co-review manuscripts.

We are delighted that the reviewers are satisfied with our revised manuscript and do not require any additional experiments or changes to the manuscript. Although Reviewer 2 remains critical of the novelty of our findings, he/she does not have any further criticism of the quality or quantity of the work. Hence, we revised the paper further to ensure that it complies of the editorial requirements of Nature Communications.

Reviewer #1 (Remarks to the Author):

The authors have sufficiently addressed my concerns.

We thank the reviewer for the careful consideration of the revised manuscript.

Reviewer #2 (Remarks to the Author):

The authors have responded to the earlier critiques with clarifications, arguments, and reorganization of figures, and some additional experiments. Although there is subjectivity in judging the impact of a study, I still do not interpret the findings as carrying the level of significance/novelty (justifying publication in Nature Comm.) that the authors emphasize. That said, I do not offer any criticism of the quality or quantity of the data. For that reason, I do not request additional revisions or further experiments.

We thank the reviewer for carefully evaluating the revised manuscript. We note that the reviewer remains unconvinced of the novelty of our findings. To date, experimentally determined structures of human telomerase have been limited to complexes containing a DNA substrate terminating in the −TTAGGG permutation and lacking an incoming dNTP.

In contrast, our study presents three experimentally determined structures of the human telomerase catalytic core bound to three distinct substrate permutations, each captured in the presence of an incoming nucleotide. These structures enable us to rationalise numerous prior biochemical and genetic observations and, importantly, reveal previously unrecognized regions within TERT and hTR that play critical roles in telomeric repeat addition. Given that telomeric repeat addition is central to telomerase function in vivo, our work substantially advances the mechanistic understanding of human telomerase activity.

Reviewer #3 (Remarks to the Author):

The authors have addressed all of my concerns in the revised manuscript.

We thank the reviewer for the careful consideration of the revised manuscript.

Reviewer #4 (Remarks to the Author):

I co-reviewed this manuscript with one of the reviewers who provided the listed reports. This is part of the Nature Communications initiative to facilitate training in peer review and to provide appropriate recognition for Early Career Researchers who co-review manuscripts.
